# Early onset senescence and cognitive impairment in a murine model of repeated mTBI

**DOI:** 10.1186/s40478-021-01190-x

**Published:** 2021-05-08

**Authors:** Nicole Schwab, YoungJun Ju, Lili-Naz Hazrati

**Affiliations:** 1 Department of Laboratory Medicine and Pathobiology, Faculty of Medicine, University of Toronto, Toronto, Canada; 2 The Hospital for Sick Children, Toronto, Canada

**Keywords:** Traumatic brain injury, Concussion, Neuroinflammation, DNA damage response, Senescence, Ageing, Neurodegeneration

## Abstract

Mild traumatic brain injury (mTBI) results in broad neurological symptoms and an increased risk of being diagnosed with a neurodegenerative disease later in life. While the immediate oxidative stress response and post-mortem pathology of the injured brain has been well studied, it remains unclear how early pathogenic changes may drive persistent symptoms and confer susceptibility to neurodegeneration. In this study we have used a mouse model of repeated mTBI (rmTBI) to identify early gene expression changes at 24 h or 7 days post-injury (7 dpi). At 24 h post-injury, gene expression of rmTBI mice shows activation of the DNA damage response (DDR) towards double strand DNA breaks, altered calcium and cell–cell signalling, and inhibition of cell death pathways. By 7 dpi, rmTBI mice had a gene expression signature consistent with induction of cellular senescence, activation of neurodegenerative processes, and inhibition of the DDR. At both timepoints gliosis, microgliosis, and axonal damage were evident in the absence of any gross lesion, and by 7 dpi rmTBI also mice had elevated levels of IL1β, p21, 53BP1, DNA2, and p53, supportive of DNA damage-induced cellular senescence. These gene expression changes reflect establishment of processes usually linked to brain aging and suggests that cellular senescence occurs early and most likely prior to the accumulation of toxic proteins. These molecular changes were accompanied by spatial learning and memory deficits in the Morris water maze. To conclude, we have identified DNA damage-induced cellular senescence as a repercussion of repeated mild traumatic brain injury which correlates with cognitive impairment. Pathways involved in senescence may represent viable treatment targets of post-concussive syndrome. Senescence has been proposed to promote neurodegeneration and appears as an effective target to prevent long-term complications of mTBI, such as chronic traumatic encephalopathy and other related neurodegenerative pathologies.

**Supplementary Information:**

The online version contains supplementary material available at 10.1186/s40478-021-01190-x.

## Introduction

Mild traumatic brain injury (mTBI) is common and can cause a broad range of debilitating symptoms, including headaches, fatigue, irritability, sleep disturbances, depression and anxiety, and attention deficits [61, 94, 101]. While some individuals recover within days to weeks after injury, others go on to have persistent long-term effects [147]. In particular, repeated mTBI (rmTBI) has been identified as a major risk factor for neurodegenerative diseases including Alzheimer’s disease (AD) [100], Parkinson’s disease (PD) [68] amyotrophic lateral sclerosis (ALS) [21, 2], fronto-temporal dementia (FTD) [126, 124], and chronic traumatic encephalopathy (CTE) [95].

Brain changes following mTBI are still under investigation, however, it is established that immediately following mTBI there is a significant increase of oxidative stress [52, 140]. This can lead to widespread DNA damage [28]. Indeed, accumulation of DNA damage has been reported in various models of mTBI [3, 123, 151] and our lab has previously shown evidence of double-strand breaks (DSBs) in human brains with mTBI history [131, 132]. Although cells throughout the body regularly encounter DNA damage from endogenous sources, such as metabolic reactive oxygen species (ROS), the cell’s DNA damage response (DDR) can become overwhelmed when facing pathological levels of damage. The DDR is a dynamic pathway which functions to restore DNA integrity following various types of lesions, involving signalling molecules, transcription factors, and repair enzymes. If the DDR becomes persistently activated in the face of persistent DNA damage, cellular senescence pathways may be activated. Cellular senescence is characterized by cell-cycle arrest, chronic inflammation via the production of senescence-associated secretory phenotype (SASP) factors, morphological abnormalities, and anti-apoptotic gene expression signatures. Senescent cells accumulate with age [23, 157, 175], neurodegenerative disease and cognitive decline [15], neuropsychiatric disorders [33], and have recently been discovered to accumulate after TBI [3, 123, 131, 132, 151].

In this paper, we use a mouse model of rmTBI to identify gene expression changes at 24 h and 7 days post-injury. We suggest that activation of the DDR occurs in the 24 h period following mTBI, which drives changes consistent with cellular senescence and early neurodegeneration by 7d post-injury.

## Materials and methods

### Animals

All experiments were approved by the Centre for Phenogenomics (TCP) Animal Care Committee. Adult (7–9 week old) male C57BL/6 mice (TCP in-house colony), housed randomly, were used in this study. Mice were kept under standard laboratory conditions with access to food and water ad libitum. Separate groups of mice were used for neurobehavioural testing including righting reflex and Morris water maze (n = 8 mice per group), NanoString gene expression analysis and histology (n = 3 mice per group), and molecular analysis with qPCR and Western Blot (n = 3 mice per group).

### Repeated mild traumatic brain injury model

Mice were randomly assigned to receive rmTBI or sham procedures. The brain injury model used in this study is a closed-skull controlled impact model. Mice received pre-operative subcutaneous injections of sustained release buprenorphine (1.2 mg/kg) and were then anesthetized with isoflurane (induced at 4% and maintained at 2.5%). Once anesthetized, mice were injected subcutaneously with lactated ringers with 5% dextrose (0.75 ml), and under the scalp with equal parts xylocaine (with epinephrine) and bupivacaine (total injection volume of 0.1 ml). Mice were placed in a stereotactic frame, preventing movement of the head, where a midline incision was made to expose the skull. The desired location (2.5 mm right of Bregma, over the right somatosensory cortex) was identified and an impact of 200 m/s was administered with an electromagnetically driven Impact One Stereotaxic Impactor (Leica, Buffalo Grove, IL) using a 5 mm metal tip, to a depth of 1.5 mm, and with a dwell time of 200 ms.

### Neurobehavioural testing

Following the impact, mice were placed in a clean recovery cage and righting reflex was assessed by placing mice on their backs and measuring the time for mice to “right” by turning over onto all four paws. This procedure was repeated for a total of three impacts with a 24-h inter-impact interval. Sham animals received all procedures without the impact. One week following the final injury, mice were subjected to neurobehavioural testing with the Morris water maze task over 4 days. Each testing day starts with the mice being left in their home cages inside of the testing room or in an anteroom for at least 30 min prior to testing, to help them acclimate to the testing environment. The maze is positioned centrally under a ceiling camera and roughly 1 m away from surrounding walls with four visual cues around the pool. The pool is filled with water to approximately 10 cm from the edge (providing mice with a clear view of external cues), heated to 25 °C, and coloured with non-toxic white paint to make the platform, placed in the centre of a target quadrant, invisible. Days 1–3 make up the training days, in which each mouse undergoes four blocks of three trials (total of 12 trials per day per mouse) with ~ 15 min of rest between trial blocks. On day 4 the platform was removed, and the time spent in the platform zone was measured.

### Statistical analysis of neurobehavioural tasks

Raw data from Ethovision (Noldus) was obtained and analyzed using the Rtrack in R, a package for reproducible and automated water maze analysis using a machine learning classifying strategy [116]. Metrics obtained from this package were latency to platform, time in goal zone, and search strategy classification.

### Animal sacrifice

Following the three impacts or sham surgeries, mice for tissue analysis were sacrificed at 1 day or 1 week after the final injury, and mice used for behavioural testing (not included in our tissue analyses) were sacrificed on the final day of behavioural testing. For NanoString analysis and histology, mice were sacrificed via transcardial perfusion, first with PBS and heparin then with 4% paraformaldehyde, under ketamine (150 mg/kg) and xylazine (10 mg/kg). Following perfusion, the brain in the skull was removed and post-fixed overnight in 4% PFA. Following post-fixation, the hemispheres were separated, and each brain half was processed and embedded in paraffin blocks. For molecular analysis, mice were sacrificed via transcardial perfusion under ketamine and xylazine with PBS and heparin. The brains from these mice were immediately dissected out of the skull and frozen in a bath of isopentane in liquid nitrogen.

### Histology

Formalin-fixed paraffin embedded (*FFPE*) blocks from the ipsilateral side of each mouse brain were used for immunohistochemistry. Sagittal blocks were cut into six micron sections and mounted on glass slides. Sections were stained with hematoxylin and eosin (H&E), glial fibrillary acidic protein (GFAP, Dako), Ionized calcium binding adaptor molecule 1 (IBA1, Abcam), amyloid precursor protein (APP, EMD Millipore 1:5000), and Lamin A/C (Santa Cruz, 1:200).

### RNA extraction and isolation

FFPE blocks from the ipsilateral side of each mouse brain were used for gene expression. Shavings from each block (containing structures spanning the entire ipsilateral hemisphere excluding cerebellum) were taken, and total RNA was extracted and isolated using the RNeasy FFPE Kit by Qiagen (Qiagen Inc., Toronto, ON, Canada) with no changes to the manufacturer’s protocol. Total RNA was quantified using the Nanodrop 2000 spectrophotometer (NanoDrop Technologies, Wilmington, DE, USA). Approximately 200 ng RNA was used from each sample for expression profiling.

### Nanostring nCounter mouse panel

Gene expression was assessed with a NanoString nCounter gene expression panel (Nanostring Technologies, Seattle, WA, USA). This panel allows multiplex gene expression analysis in fixed tissue of 770 genes broadly involved in pathways involved in inflammation, neurobiology, neuropathology, and metabolism and stress. For data normalization processes, this panel includes 13 internal references genes. A list of all target genes used in this study can be found in Additional files 1 and 2, where ratio data can also be found.

### Statistical analysis of nanostring data

Raw data was collected and assessed using NanoString Technologies nSolver Analysis Software 4.0. Briefly, quality control was performed using default settings, normalization was performed using the geometric mean of positive control counts and housekeeping normalization. Fold change (FC) ratios were calculated between rmTBI and shams and a two-tailed t-test was performed to determine FC ratio significance. All fold change data for 24 h post-injury can be found in Additional file 1, and for 7d post-injury can be found in Additional file 2. Heat map clusters were generated using Euclidian distance and average linkage methods and all gene expression figures were generated using nSolver. Gene ontology analysis was performed using the g:Profiler open-source g:GOSt software. Specifically, functional terms were retrieved from the Gene Ontology (GO) database containing terms for biological process, molecular function, and cellular component. Pathway analysis used the Kyoto Encyclopedia of Genes and Genomes (KEGG), Reactome, and WikiPathways (WP) databases. Terms unrelated to the brain were omitted, and the top 20 results from each group (i.e. from each biological process, molecular function, cellular component, and pathway groups). Statistical analysis was performed using the g:SCS method in g:Profiler, a default method for multiple testing corrections. Statistical threshold was set to adjusted *p* ≤ 0.05.

### Western Blot and quantitative real-time PCR

For Western Blot (WB) the ipsilateral hemisphere, excluding cerebellum and brainstem, was homogenized in RIPA buffer (Millipore) containing a protease and phosphatase inhibitor cocktail (Thermofisher). The supernatants were harvested after centrifugating at 12,000*g* for 20 min. Protein concentration was measured using a protein assay kit (Biorad). Protein samples were loaded in 10% polyacrylamide gels (Biorad) and transferred to a nitrocellulose membrane (Biorad). Membranes were blocked in 5% BSA of tris buffered saline with 0.05% tween-20 (TBS-T) for 2 h and incubated with primary antibodies for 2 h at room temperature or overnight at 4 °C. HRP conjugated secondary antibodies [Sigma, anti-mouse (A9044, 1:10,000) and rabbit (A0545, 1:20,000)] were employed. Membranes were visualized by the Odyssey Fc imaging system (LI-COR). Primary antibodies were beta-tubulin (Sigma, T7816, 1:20,000), 53BP1 (Novus biologicals, NB100-304, 1:10,000), DNA2 (Thermofisher, PA5-68167, 1:1000), Bcl-w (Cell signaling technology, #2724, 1:1000), and p53 (Santa cruz biotechnology, sc-126, 1:1000).

For qPCR: the ipsilateral hemisphere, excluding cerebellum and brainstem, was homogenized in Trizol (Invitrogen) for total RNA isolation. RNA quality was determined using a Nanodrop one (Thermofisher). 1–2 µg of total RNA was reverse-transcribed using the cDNA synthesis kit (SuperScript VILO (Invitrogen)) according to the manufacturer’s protocol. qPCR was performed by QuantaStudio 3 system (Applied Biosystems) using SYBR select master mix (Thermofisher). The information of primers is in Additional file 3: Table S1. Statistical analysis for both WB and qPCR data was performed using unpaired student t-test.

## Results

### rmTBI mice show loss of righting reflex indicative of a mild injury

**Fig. 1:**
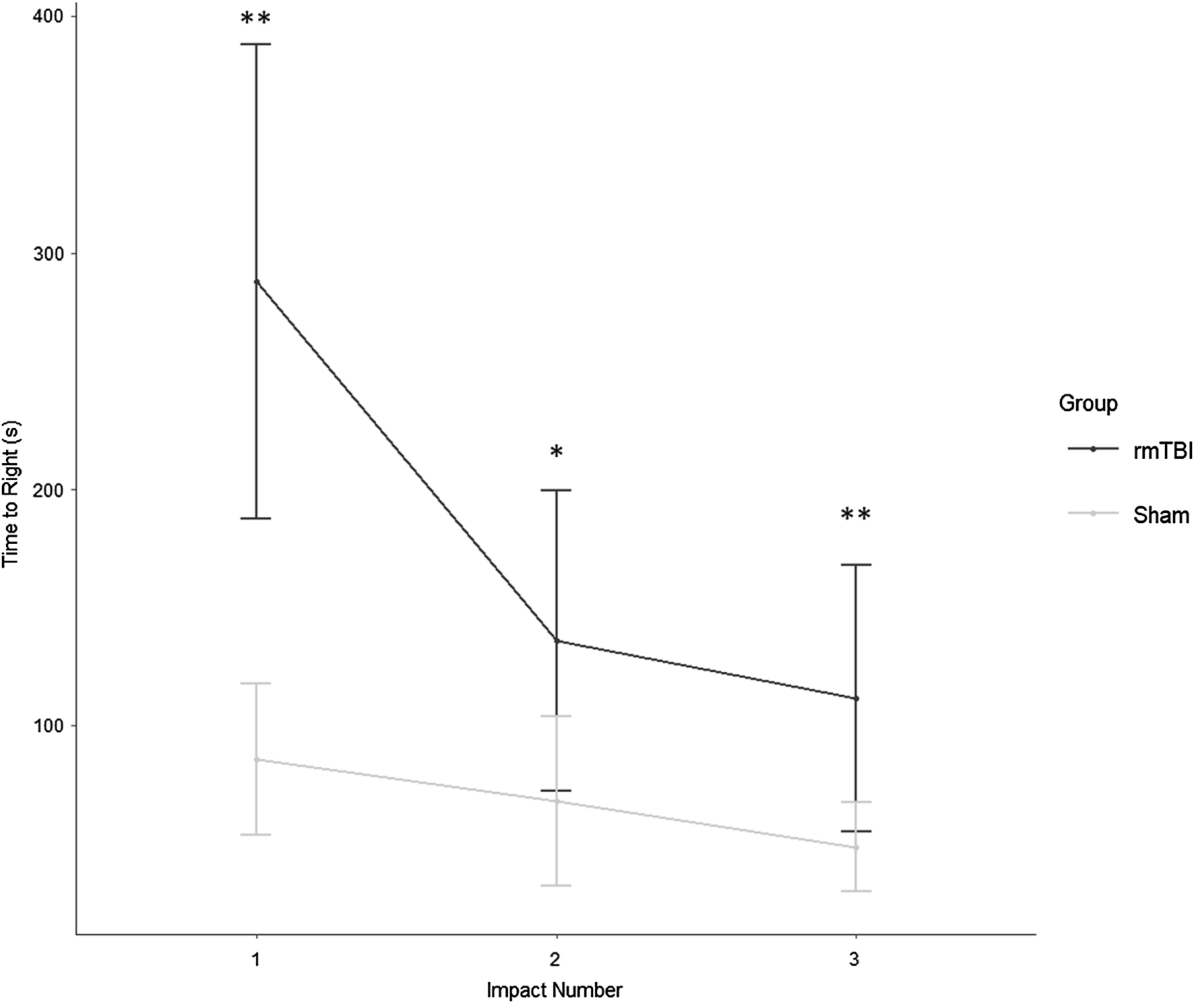
The duration of loss of righting reflex in rmTBI was significantly longer than in sham-treated mice after each impact (*p* < 0.001 main effect of injury status, repeated measures ANOVA), and decreased significantly with increased impact number (*p* < 0.001, interaction between injury status and impact number, repeated measures ANOVA). Righting reflex was under 15 min for all mice, supportive of a mild injury

Loss of righting reflex was evident in the rmTBI mice (Fig. 1). rmTBI mice took an average of 186.0 s longer to right after the first impact (*p* = 0.001), 61.7 s longer after the second impact (*p* = 0.02), and 63.1 s longer after the third impact (*p* = 0.01). The longest righting reflex recorded remained under 15 min (900 s), which is indicative of a mild injury [106].

### Spatial learning and memory impairment 1 week after rmTBI

**Fig. 2:**
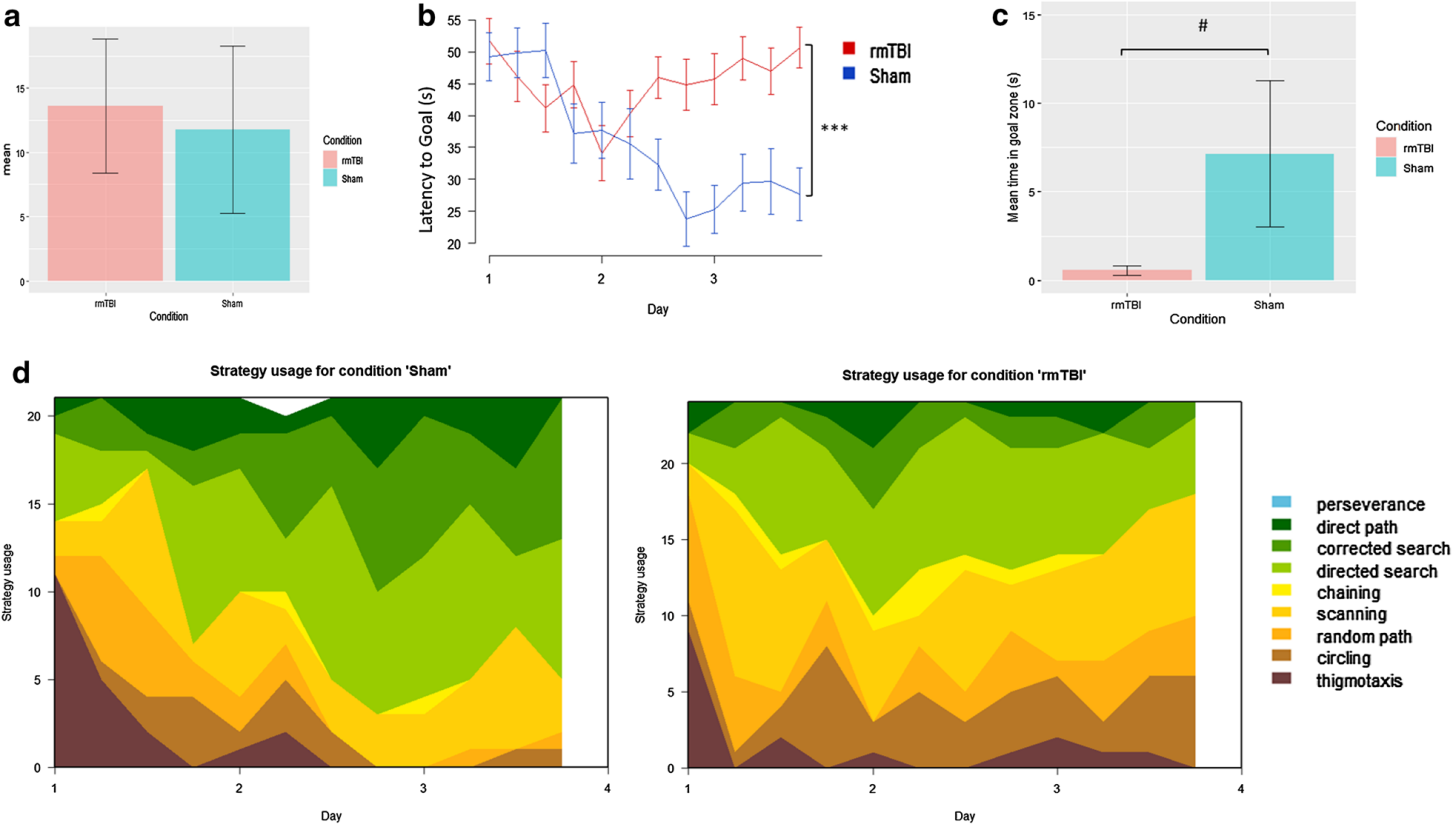
rmTBI mice show evidence of spatial learning and memory impairment at 1 week post-injury. **a** rmTBI mice swam an average of 1.8 cm/s faster than sham mice. **b** Across three training days rmTBI mice had a significantly increased latency to find the platform compared to shams (*p* < 0.001, main effect of injury status, repeated measures ANOVA). **c** In the probe test rmTBI mice spent less time in the platform zone compared to shams (*p* = 0.55, Mann–Whitney U Test). Analysis of search strategies with Rtrack revealed no significant differences one training day one, however rmTBI mice used significantly different search strategies by training days two (χ^2^ = 6.2, *df* = 2, *p* = 0.05) and three (χ^2^ = 33.9, *df* = 2, *p* < 0.0001) with rmTBI mice utilizing less allocentric strategies than shams by the end of training

rmTBI mice and sham mice differed slightly in their average swim speed, with rmTBI mice swimming 1.8 cm/s faster compared to shams on average (Fig. 2a). Across three training days, rmTBI mice spent significantly longer searching for the hidden platform (latency to goal) compared to shams at 1 week post-injury with a main effect of injury (*p* = 0.0001, repeated measures ANOVA), a main effect of training day (*p* = 0.047, repeated measures ANOVA) and a significant interaction between injury status and training day (*p* = 0.001, repeated measures ANOVA) indicating that while the shams improved their performance over time whereas the rmTBI mice did not (Fig. 2b). In the probe test rmTBI mice spent less time in the goal zone compared to shams, which was almost significant (*p* = 0.55, Mann–Whitney U Test) (Fig. 2c).

Search strategy use was analyzed using a machine learning classifier package in R called RTrack [116] (Fig. 2d). Three main categories of search strategies were assessed. Non-goal-oriented strategies include thigmotaxis, circling, and random paths, indicating that the mouse is not attempting to reach the goal but is instead motivated to escape the pool. Procedural strategies, including scanning and chaining, indicating that the mouse is aware of a goal, but is using egocentric measures to find it. Last, allocentric strategies including direct searches, corrected searches, and direct paths indicate that the mouse is orienting itself based on cues around the room to find the goal.

On the first training day the use of search strategies was not significantly different between rmTBIs and shams (χ^2^ = 3.4, *df* = 2, *p* = 0.18). However, rmTBI and sham mice utilized significantly different search strategies on training day two (χ^2^ = 6.2, *df* = 2, *p* = 0.05) and three (χ^2^ = 33.9, *df* = 2, *p* < 0.0001). Across the trials from training day one to training day three rmTBI mice only increased the use of allocentric strategies by 4.2% by the end of training, whereas shams had increased the use of allocentric strategies by 36.9%. Similarly, rmTBI mice remained stagnant using 65.6% non-goal oriented and procedural strategies by training day three, whereas shams only used these strategies 26.2% of the time. These results suggest that rmTBI mice have significant deficits in their spatial learning and memory retention, specifically for the ability to learn and use spatial cues and rely instead on mostly egocentric measures to complete the task.

### Histological assessment of brains

**Fig. 3:**
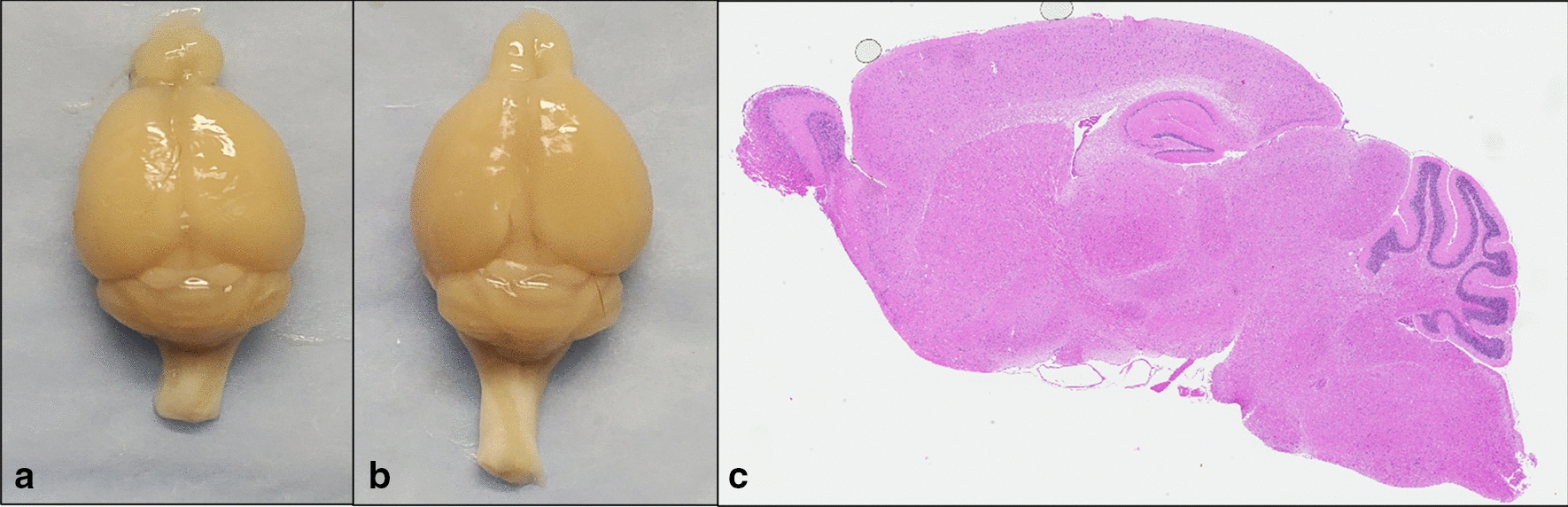
Gross morphology of the mouse brain following Sham (**a**) and rmTBI (**b**) procedures. H&E staining of the rmTBI brain (**c**) reveals no visible lesion induced by the injury model, indicating a mild injury has been elicited

rmTBI brains showed normal gross morphology, with an absence of any visible lesion at the region of impact and did not visibly differ from sham brains (Fig. 3). Parasagittal histological section of the brain stained with Hematoxylin and eosin show absence of structural damage such as contusion or hemorrhage in ipsilateral cortex and underlying structures.

### Gliosis, microgliosis, axonal damage

**Fig. 4:**
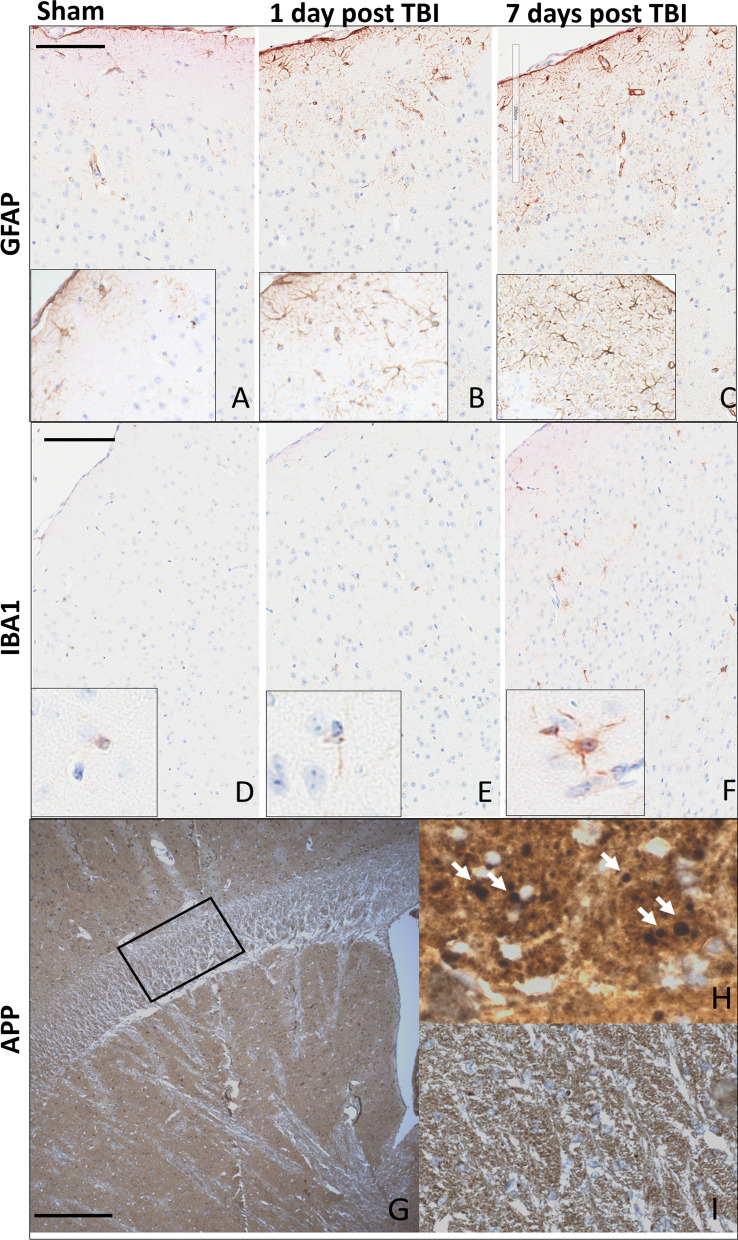
rmTBI brains have increased expression of GFAP at 1 day (**b**) and 7 days (**c**) post-injury, compared to shams (**a**) in the impact region of the isocortex, indicating gliosis. Microglial activation, shown with Iba1 staining, can be seen in rmTBI brains at both 1 day (**e**) and 7 days (**f**) post-injury compared to shams (**d**). At 7 days post-injury rmTBI mice showed evidence of axonal damage by the presence of APP positive spheroids in the subcortical white matter (**h**), which was not present in shams (**i**, low power view in **g**). Scale bars: 0.3 mm in **a**–**f**, 0.1 mm in **g**, 0.05 mm in **h**, 0.08 mm in **i**

**Fig. 5:**
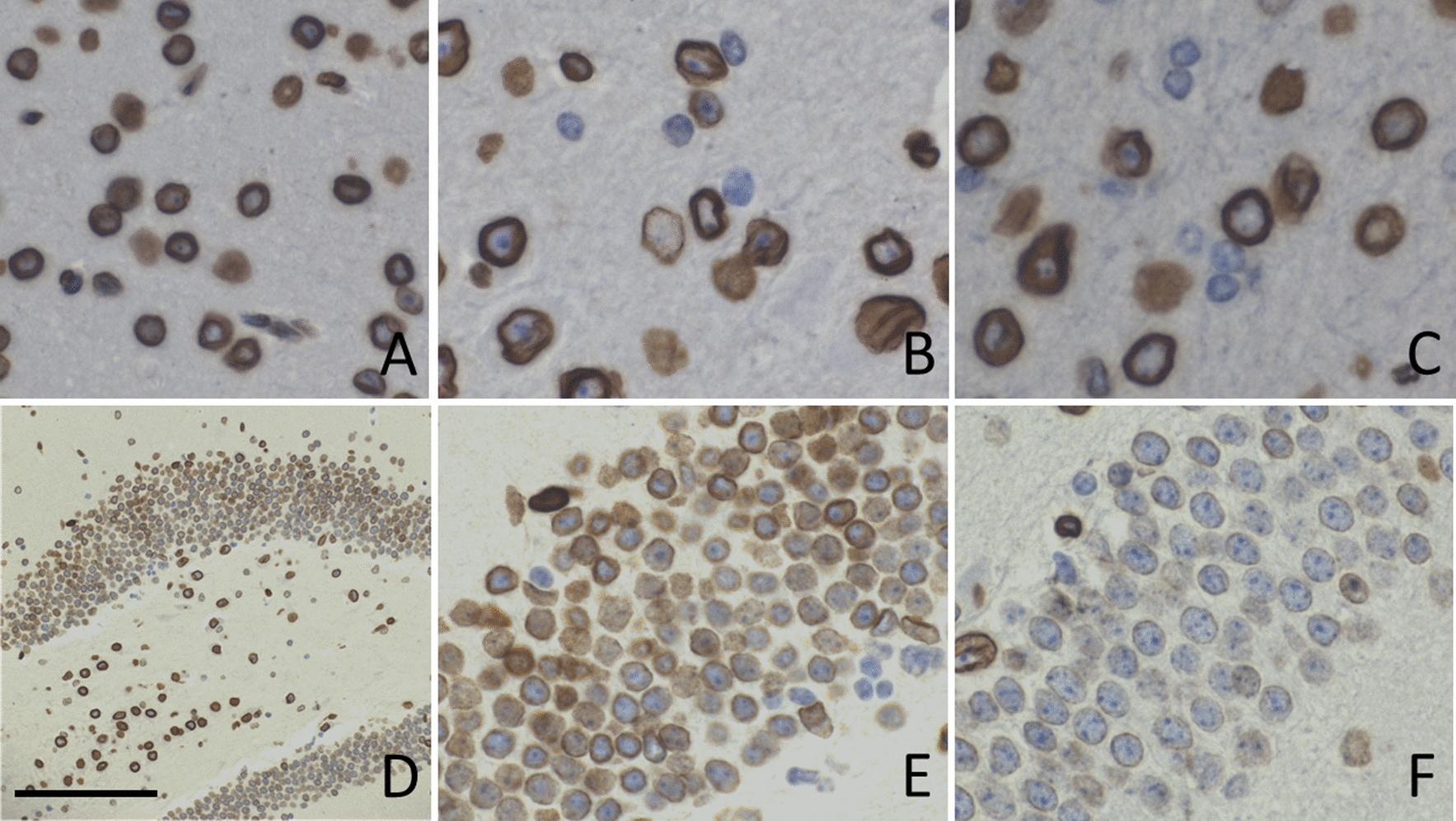
Lamin A/C expression on the nuclear membrane of glial cells was reduced at 1 day post injury in the cortex (**b**) and in neurons of the dentate (**e**), and this loss was exacerbated by 1 week post-injury in the cortex (**c**) and dentate (**f**) compared to shams (**a**, **d**). Arrows in **b** and **c** point to glial cells that have lost lamin expression. Scale bar: 150 μm in **a**–**c**, 300 μm in **d**, 120 μm in **e**, **f**

Histology revealed increased expression of GFAP and Iba1 at 1 day post-injury (Fig. 4b, e) and 1 week post-injury (Fig. 4c, f) on the side receiving the impact compared to shams (Fig. 4a, d). This suggests the presence of incremental gliosis and microglial activation, two well-researched and reproducible repercussions of the mild injury model used in this study [9]. Similarly, evidence of axonal damage after injury by increased APP staining was present at 7 days (Fig. 5b) after injury. These findings, along with a lack of gross morphology and the loss of righting reflex in injured animals, are supportive of eliciting a mild injury. Additionally, Lamin A/C (Fig. 5) immunohistochemistry shows loss of expression on the nuclear membrane of a subset of glial cells in the cortex and decreased expression in the dentate nucleus of the hippocampus. Loss of lamin A/C is associated with the induction of cellular senescence through DNA-damage sensing pathways [45].

### Activation of the DNA damage response 24 h after rmTBI

**Fig. 6:**
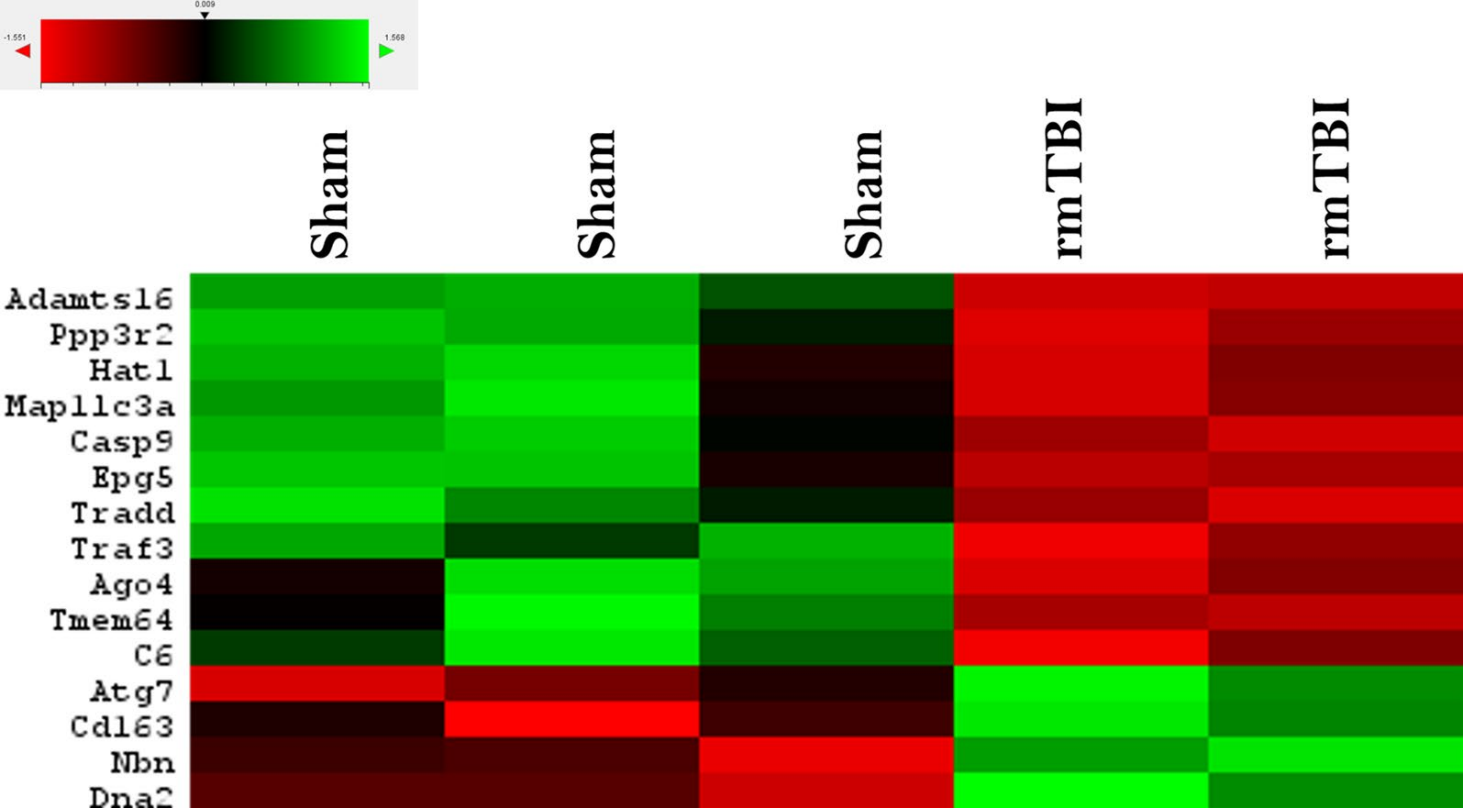
Overview of significantly (*p* ≤ 0.05) differentially expressed genes between rmTBI and sham mice at 24 h post-injury

**Fig. 7:**
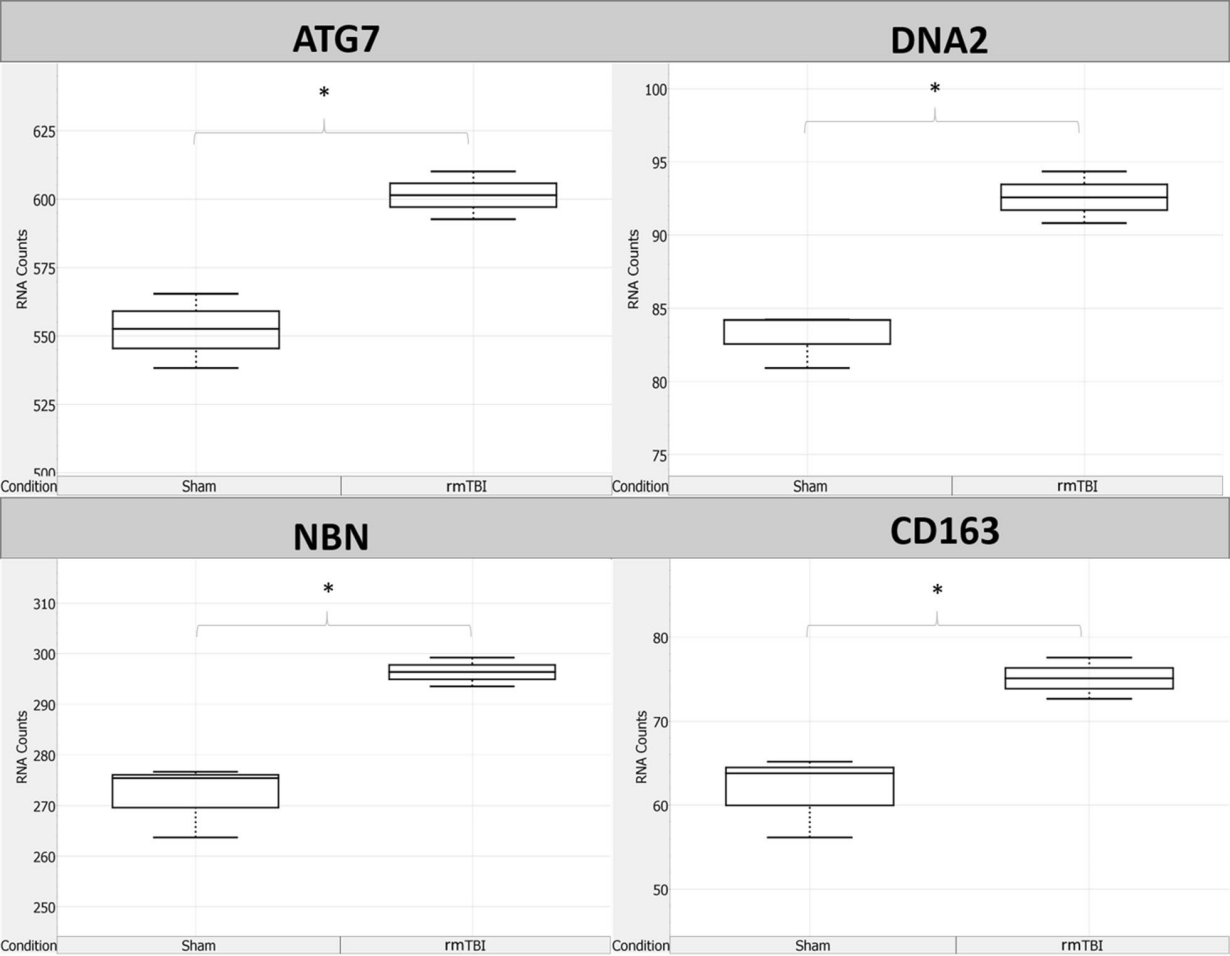
Significantly (*p* ≤ 0.05) upregulated genes in rmTBI mice compared to shams at 24 h post-injury. Data are expressed as normalized RNA count number for shams and rmTBI. The median value is represented by the horizontal line within the box, defined by the first and third quartiles, and tails represent 1.5 × the interquartile range

24 h following the final injury, there were a total of 15 genes significantly changed between rmTBI mice and shams in the ipsilateral hemisphere (Fig. 6). Of these changes, 4 genes were significantly upregulated (Fig. 7). Three of these four genes encode for DNA damage repair proteins in the DDR: ATG7 (FC = 1.09, *p* = 0.03), DNA2 (FC = 1.11, *p* = 0.04), and NBN (FC = 1.09, *p* = 0.02). ATG7 is an autophagy-related protein [136] which can modulate p53-dependent cell cycle pathways during prolonged stress [78] and plays a critical role in homology-directed repair (HDR) of DSBs [46]. Similarly, DNA2 encodes a HDR DNA repair protein for repair of DSBs [62] and NBN encodes a non-homologous end joining (NHEJ) DNA repair protein for repair of DSBs [158]. Together, the significant upregulation of these three genes in rmTBI mice compared to shams indicates the presence of DSBs at this timepoint and the activation of the DDR. The fourth gene found to be upregulated 24 h post-injury was CD163 (FC = 1.22, *p* = 0.04), which encodes a cell surface marker of activated M2 phenotype macrophages [71], suggesting an increase in the presence and activation of macrophages 24 h post-injury.

**Fig. 8:**
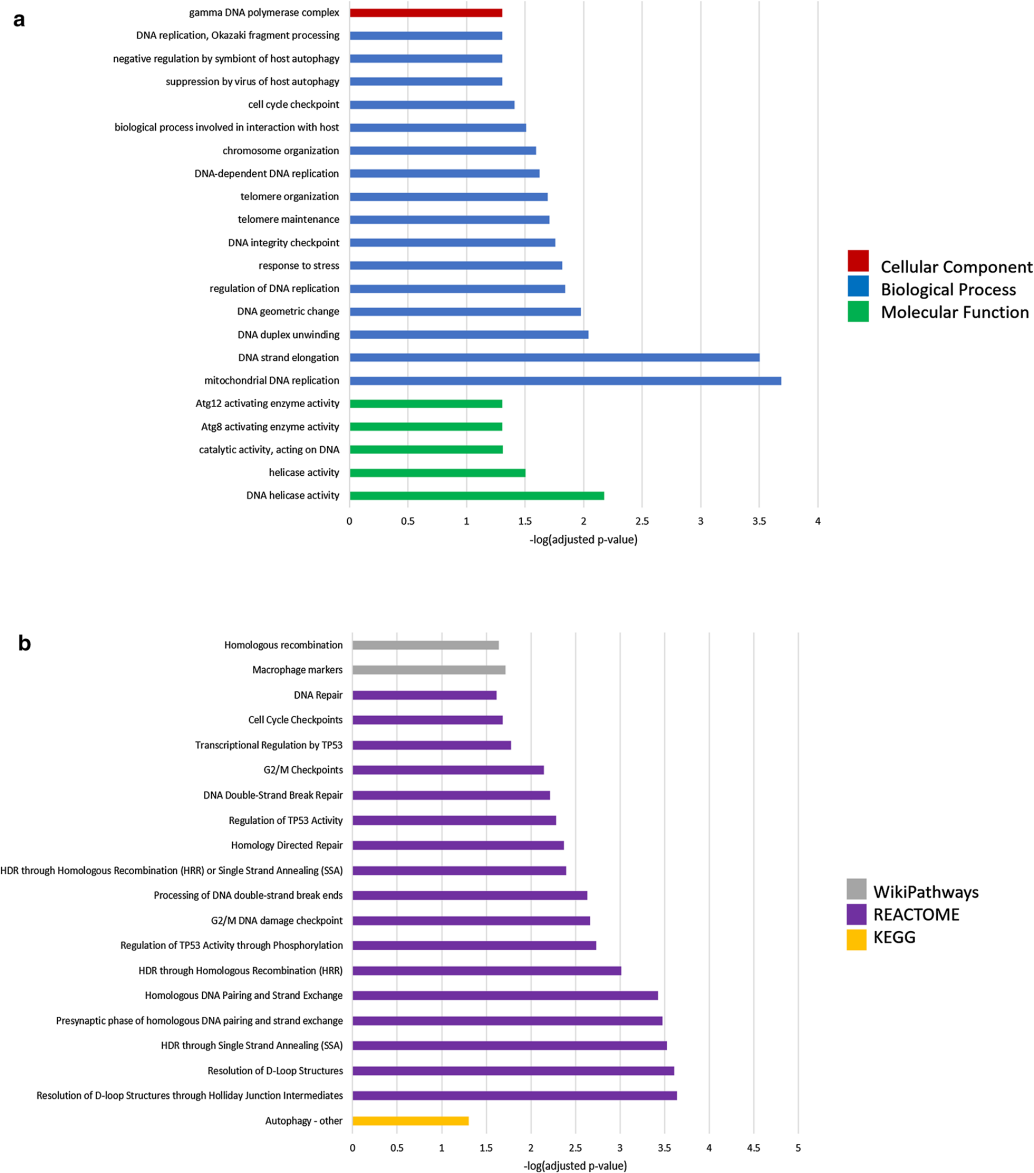
Gene set enrichment analysis of genes significantly (*p* ≤ 0.05) upregulated 24 h post-injury. **a** Gene ontology (GO) functions significantly enriched include DNA damage response pathways and **b** pathways significantly enriched include the DNA damage response and cell-cycle checkpoints

Gene ontology of genes significantly upregulated 24 h post-injury revealed enrichment of 22 GO terms (Fig. 8a). Five molecular terms were enriched: DNA helicase activity, helicase activity, catalytic activity acting on DNA, ATG8 activating enzyme, and ATG12 activating enzyme. These suggest enrichment of DNA repair pathways and autophagy signalling. Sixteen biological process terms were enriched, including autophagy, cell-cycle checkpoint, chromosome organization, DNA replication, integrity, elongation, and unwinding, response to stress, and telomere maintenance. These terms support the activation of the DDR at this timepoint, particularly the induction of cell-cycle arrest and DNA regulating and maintenance pathways to begin repair processes. One cell component term was enriched, gamma DNA polymerase complex, further supporting activation of DNA repair processes at this timepoint.

Pathway analysis of upregulated genes at 24 h post-injury revealed enrichment of 20 terms (Fig. 7b). Two terms from the WP database were enriched: homologous recombination and macrophage markers. Sixteen terms from the Reactome database were enriched, encompassing DNA repair (including DNA double-strand break repair, homology-directed repair, and resolution of D-loop structures) and cell-cycle checkpoints. Lastly, one term from the KEGG database, autophagy-other, was enriched. These terms support the activation of the DNA damage response, particularly towards DSBs, at this timepoint.

**Fig. 9:**
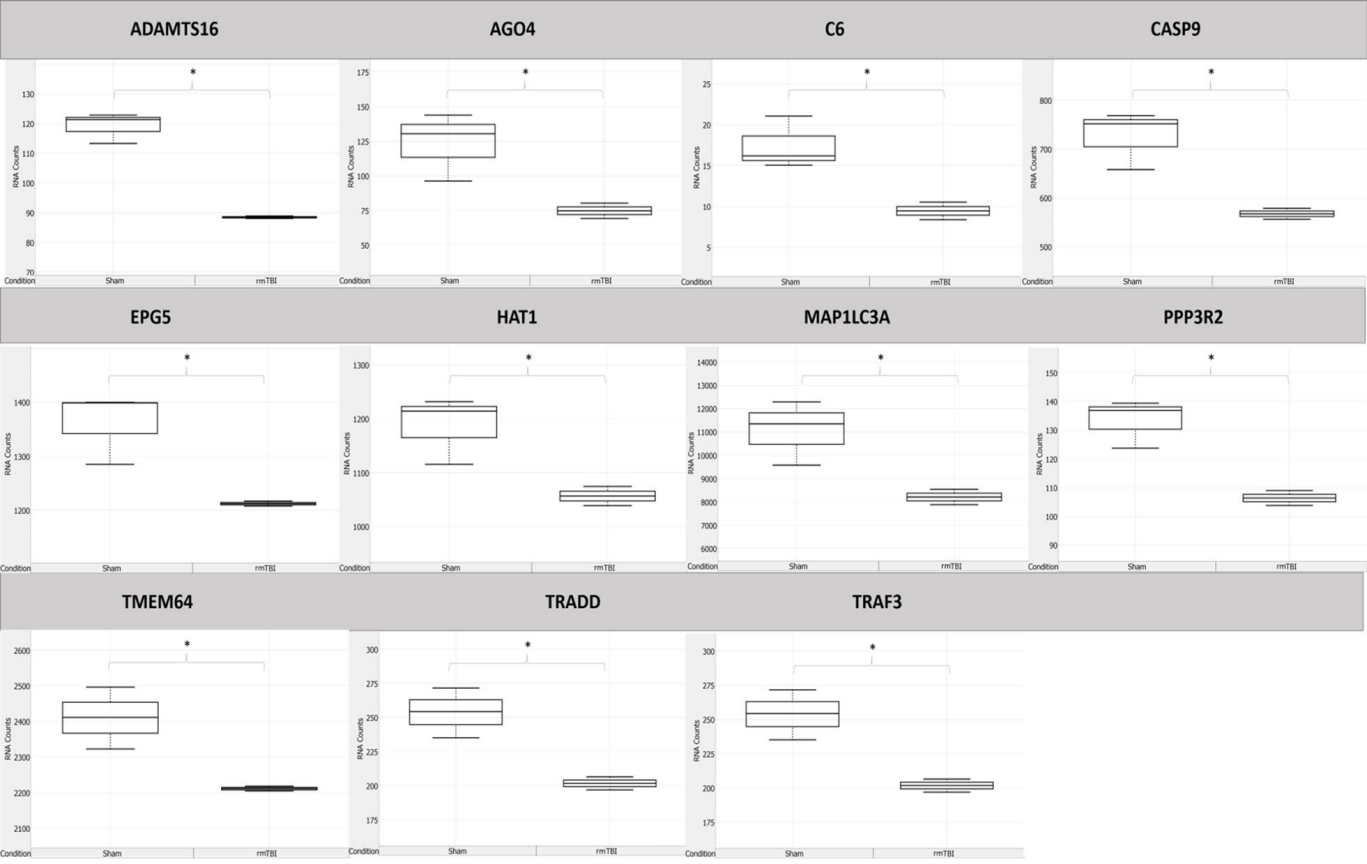
Significantly (*p* ≤ 0.05) downregulated genes in rmTBI mice compared to shams at 24 h post-injury. Data are expressed as normalized RNA count number for shams and rmTBI. The median value is represented by the horizontal line within the box, defined by the first and third quartiles, and tails represent 1.5 × the interquartile range

11 genes were significantly downregulated in the rmTBI ipsilateral hemisphere 24 h post-injury (Fig. 9), representing changes in major signalling pathways including DNA methylation, apoptosis, autophagy, TNF-associated signalling, and calcium signalling.

AGO4 (FC = −1.63, *p* = 0.04) plays a critical role in the RNA-directed DNA methylation complex [19], supporting altered DNA methylation patterns 24 h post-injury. CASP9 (FC = −1.28, *p* = 0.026) is an initiator in the apoptotic pathway, and so its downregulation is both anti-apoptotic and protective against TNF-receptor-activated apoptosis [93]. EPG5 (FC = −1.12, *p* = 0.05) is an autophagy-related gene which is essential for the formation of degradative autolysosomes [162]. EPG5 is reduced in rodent models of ischemia [177] and mediates neuronal survival and normal functioning [179]. Furthermore, 7 single-nucleotide polymorphisms (SNPs) in EPG5 have been associated with AD, and 8 SNPs have been associated with an earlier age of onset of AD [161], suggesting that alterations in EPG5’s expression may confer susceptibility to neurodegeneration. HAT1 (FC = −1.12, *p* = 0.05) is a cytoplasmic histone deacetylase, for which loss is associated with early onset ageing and mitochondrial defects [105], and for which expression is reduced under treatment with hypoxic agents [159]. MAP1LC3A (FC = −1.34, *p* = 0.04) is a microtubule associated protein which mediates the interaction between microtubules and the cytoskeleton [22] and is a key protein in the autophagy pathway. PPP3R2 (FC = −1.25, *p* = 0.02) is a subunit of calcineurin, a critical calcium and calmodulin-dependent serine/threonine protein phosphatase involved in a wide variety of brain functions including neurotransmitter release, receptor functioning, signal transduction systems, cell death pathways, and gene expression changes [41, 49]. It has been previously reported that calcineurin subunit 2 expression decreases following TBI [165, 166] and its decrease may confer susceptibility to AD [120, 156]. Indeed, calcineurin is hypothesized to be a modulator of tau in the presence of amyloid deposition and its downregulation may accelerate AD progression [120, 156]. TRADD (FC = −1.26, *p* = 0.02) encodes TNFRSF1A-associated death domain and is a mediator of the TNF-receptor-activated apoptosis [20]. In addition, deficiency of TRADD has been shown to result in the accumulation of DSB foci [75]. TRAF3 (FC = −1.12, *p* = 0.03) is a TNF-receptor-associated factor which is critical for NF-kappaB activation and cell death pathways [60]. TRAF3 has been shown to reduce expression with age [82]. Lastly, 2 significantly downregulated genes at this timepoint, ADAMTS16 (FC = −1.35, *p* = 0.006) and TMEM64 (FC = −1.09, *p* = 0.05), do not yet have established functions in the brain. Taken together, this expression profile suggests that 24 h post-injury there is significant reduction in expression of major cell death signalling pathways, possibly conferring vulnerability to subsequent brain damage and future neurodegenerative pathologies. C6 (FC = −1.84, *p* = 0.04) encodes complement factor 6, a late complement component which forms the membrane attack complex. Deficiency in this component in small mammals has been shown to cause a progressive neurological syndrome characterized by multifocal motor neuropathy and severe axonal degeneration [44].

**Fig. 10:**
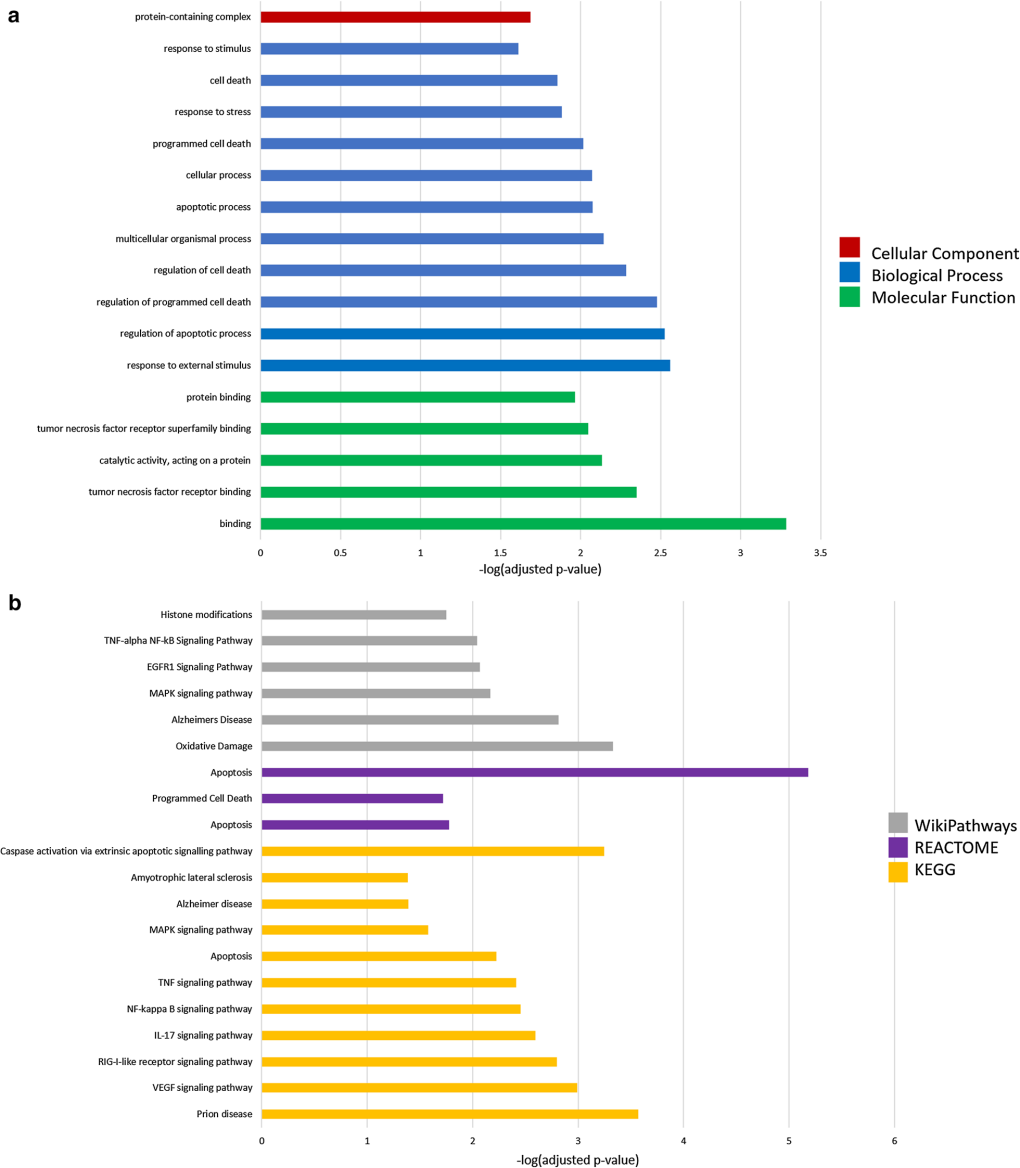
Gene set enrichment analysis of genes significantly (*p* ≤ 0.05) upregulated 24 h post-injury. **a** Gene ontology (GO) functions and **b** pathway analysis suggest inhibition of cell death pathways and altered metabolic function

Gene ontology analysis of genes significantly downregulated 24 h post-injury revealed enrichment of 17 GO terms (Fig. 10a). Five molecular function terms were enriched: protein binding, tumor necrosis factor receptor superfamily binding, catalytic activity acting on a protein, tumor necrosis factor receptor binding, and binding. Eleven biological process terms were enriched, including response to stimulus, regulation of cell death, response to stress, and regulation of apoptotic process. One cellular component term, protein-containing complex, was enriched. Enrichment of these terms in the downregulated genes at 24 h post-injury suggests that TNF-receptor-mediated activity, cell death including apoptosis, and the cellular stress response are reduced at this timepoint.

Pathway analysis of downregulated genes at 24 h post-injury revealed enrichment of 19 terms (Fig. 10b). Seven terms from the WP database were enriched: histone modifications, TNF-alpha-NF-kB signalling, EGFR1 signalling, MAPK signalling, Alzheimer’s disease, oxidative damage, and apoptosis. Three terms from the Reactome database were enriched: programmed cell death, apoptosis, and caspase activation via extrinsic apoptotic signalling. Lastly, ten terms from the KEGG database were enriched including ALS, AD, MAPK signalling, apoptosis, TNF signalling, NF-kappa-B signalling, IL-17 signalling, RIG-I-like receptor signalling, VEGF signalling, and Prion disease. This analysis suggests inhibition of cell death and major energy-dependent signalling pathways 24 h post-injury, consistent with analyses of up-regulated genes at this timepoint showing activation of the DNA damage response.

### Cellular senescence, neurodegeneration, and signalling alterations 7 days after rmTBI

**Fig. 11:**
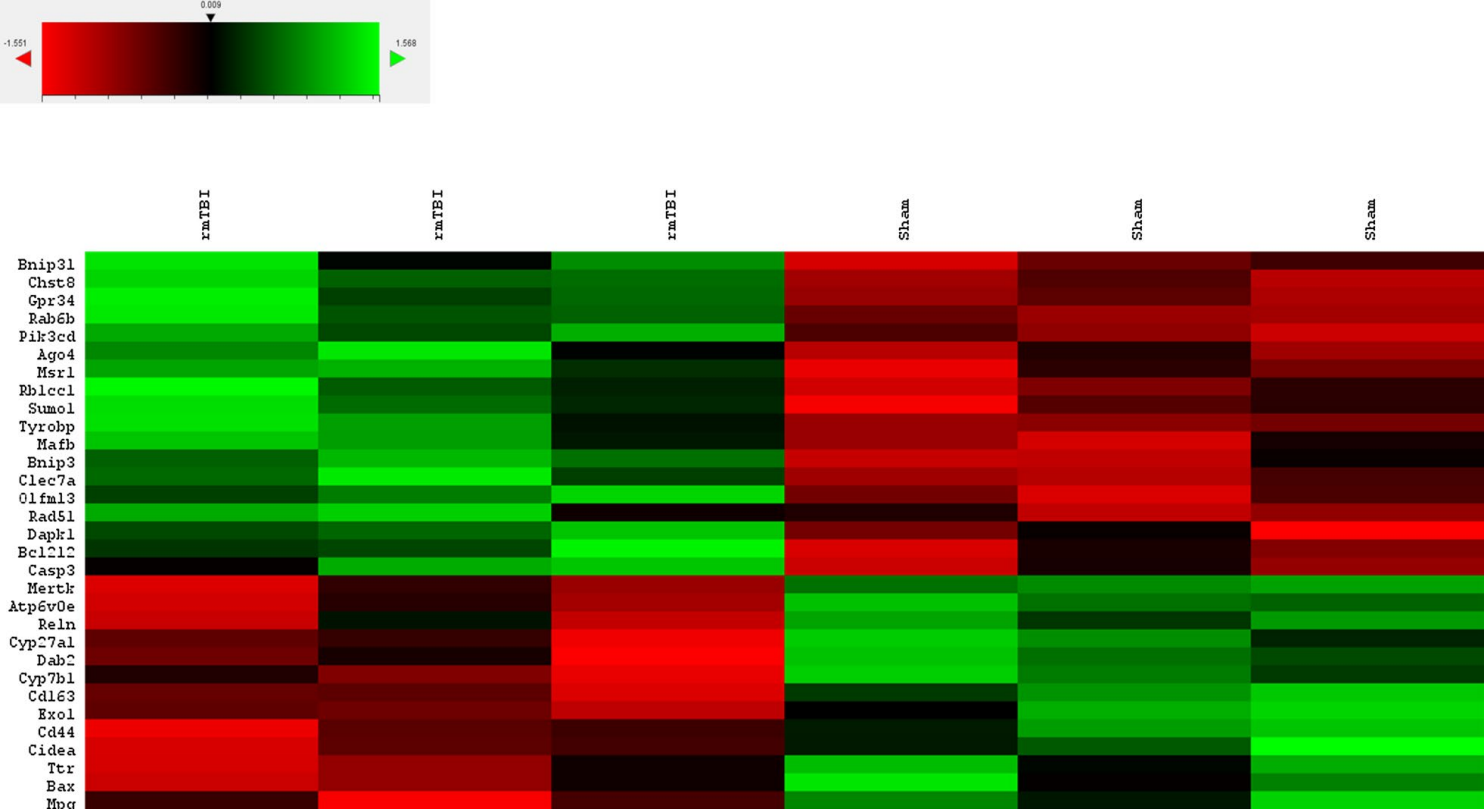
Overview of significantly (*p* ≤ 0.05) differentially expressed genes between rmTBI and sham mice at 7d post-injury

**Fig. 12:**
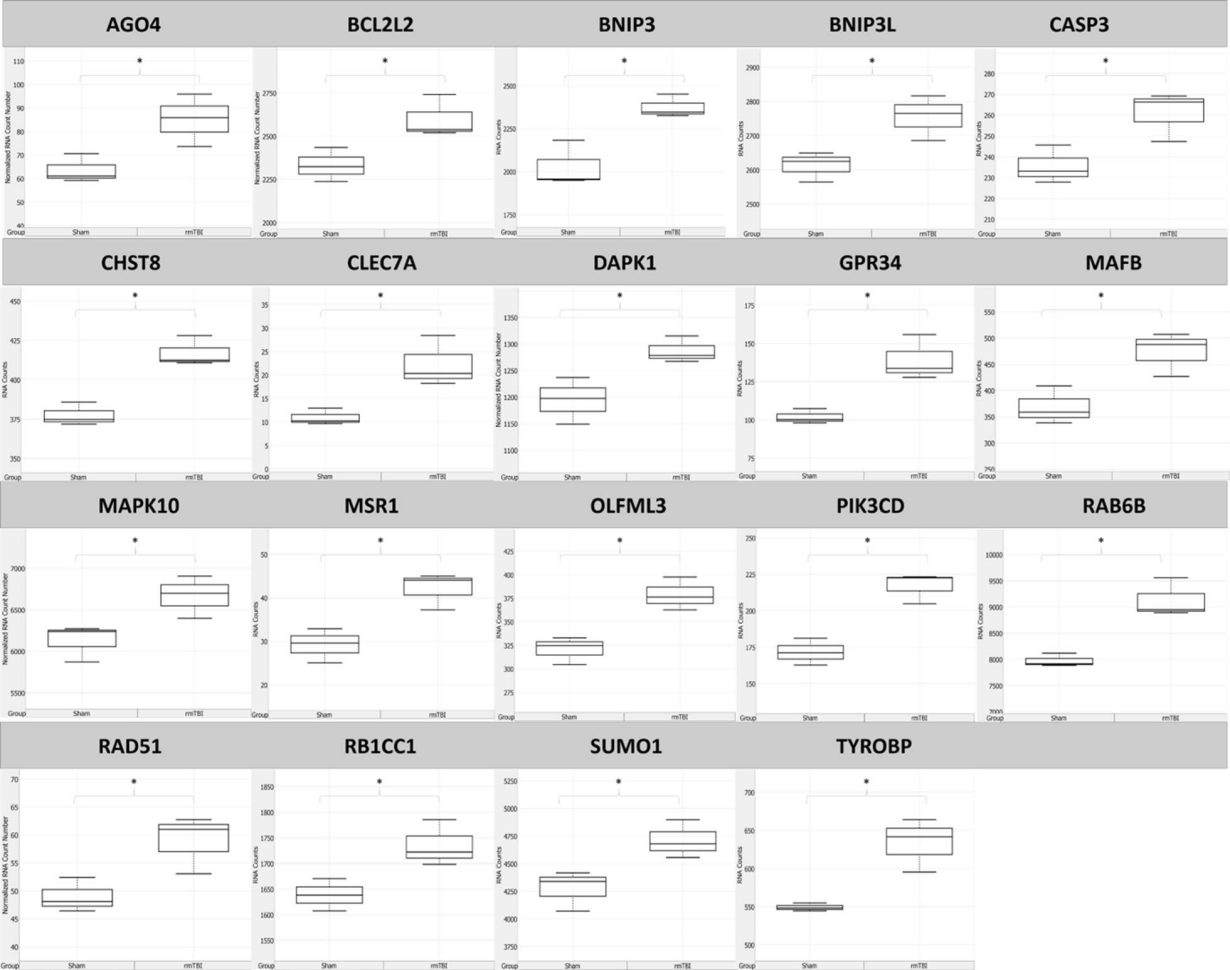
Significantly (*p* ≤ 0.05) upregulated genes in rmTBI mice compared to shams at 7d post-injury. Data are expressed as normalized RNA count number for shams and rmTBI. The median value is represented by the horizontal line within the box, defined by the first and third quartiles, and tails represent 1.5 × the interquartile range

7 days after rmTBI there were a total of 32 genes significantly changed between rmTBI mice and shams in the ipsilateral hemisphere (Fig. 11). 19 genes had significantly increased expression levels (Fig. 12), suggesting the induction of cellular senescence, activation of neurodegenerative pathways, and changes in large signalling pathways, particularly to cell death pathways, mitophagy, autophagy, metabolism, and oxidation and reduction reactions.

AGO4 (FC = 1.33, *p* = 0.04) is a member of the Argonaute family which plays a role in DNA methylation and miRNA maintenance [19]. Although relatively little is known about the role of AGO4 compared to the other known members of this protein family, for example AGO2 regulates gene expression silencing in the context of cellular senescence [6, 8], it is thought to be specific for gene expression silencing [97]. It is unclear what role AGO4 plays in the brain, but it likely plays a role in gene expression signatures perhaps in senescent cells. CASP3 (FC = 1.11, *p* = 0.04) encodes caspase 3, an executioner in the apoptosis pathway when it is proteolytically cleaved from its pro- form. CASP3 was significantly increased 7d post-injury, suggesting some level of apoptosis occurring at this timepoint. Although caspase 3 is thought to be inhibited by cellular senescence [117], it paradoxically increases expression with ageing and has even been suggested as an initiating factor for cellular senescence [152].

Several genes within the BCL2 pro-senescence signalling pathway were increased at this timepoint. BCL2L2 (FC = 1.11, *p* = 0.04) encodes the anti-apoptotic and pro-senescence BCL-W protein [173]. Increased expression of BCL2L2 is associated with neurofibrillary tangle pathology in AD [182], and it promotes the expression of senescence-associated beta-galactosidase, p53, p21, and p16 which are all hallmark features of cellular senescence [24, 54]. Inhibition of BCL2L2 results in direct elimination of senescent cells [173], supporting the role of this protein in the induction and maintenance of cellular senescence. In relation to BCL2L2, BNIP3 (FC = 1.17, *p* = 0.04) encodes BCL2 interacting protein 3, which is increased in hypoxic-ischemic-encephalopathy [18] and physiological ageing [31], and has been shown to increase in association with cellular senescence especially in cells over-expressing p21 and p16 [16]. Similarly, BNIP3L (FC = 1.05, *p* = 0.04) encodes BCL2 interacting protein 3 like. Although BNIP3L is typically thought of as pro-apoptotic [181], anti-apoptotic roles have been recently discovered, particularly in reactive astrocytes during ALS disease progression [35]. Similarly, BNIP3L has been shown to increase in senescent cells to direct mitochondria to autophagosomes for degradation [174]. RAD51 (FC = 1.2, *p* = 0.05) encodes a DNA repair enzyme which associate with gH2AX foci [103]. Together, these four genes suggest increased expression of cellular senescence pathways 7d post-injury.

In addition to the above changes reflecting cellular senescence, several of the significantly increased genes at this timepoint have been associated with neurodegenerative phenotypes and diseases. CHST8 (FC = 1.1, *p* = 0.005) encodes the enzyme carbohydrate sulfotransferase 8. This enzyme has been suggested to be enriched in AD pathology progression [144] and increased in the hippocampus of AD brains [64]. CLEC7A (FC = 2.02, *p* = 0.02) is a disease-associated microglia gene which has been shown to increase after TBI in mice [19], in a mouse model of AD [38, 56], and in aged microglia associated with neurodegenerative disease [135]. DAPK1 (FC = 1.08, *p* = 0.05) is a calcium-dependent serine/threonine kinase that is highly expressed in the cortex in AD [167], after ischemic damage [154], and in physiological ageing where it has been suggested to play a role in the decay of learning and memory with age [51]. DAPK1 has been found to phosphorylate tau protein [118], suggesting that it possibly plays a functional role in the progression of AD-related pathology. GPR34 (FC = 1.36, *p* = 0.002) encodes an inhibitory G-protein coupled receptor found on microglia which is overexpressed in regions of the brain affected by neurodegeneration in both humans and mice [10]. Although its functional importance remains unclear, GPR34 is also found to increase in aged human brains and has been postulated as a possible risk factor for AD [110]. MAFB (FC = 1.29, *p* = 0.03) is a transcriptional regulator of mature microglia which is upregulated during multiple sclerosis disease progression [81] and found to inhibit apoptosis of macrophages [89]. MAFB positively regulates the expression of MSR1 (FC = 1.45, *p* = 0.002) [137], which was also significantly increased at this timepoint. MSR1 encodes a macrophage scavenger receptor, and it is increased in mouse models of AD [128] where it plays a role in amyloid beta uptake [138]. OLFML3 (FC = 1.18, *p* = 0.01) encodes a secreted extracellular matrix glycoprotein which is highly linked to AD pathogenesis in brain tissues where it correlates with amyloid beta levels, and in the cerebrospinal fluid (CSF) where it is considered a potential biomarker of AD [160]. OLFML3 has also been identified with single cell RNA sequencing as a senescence-associated microglia gene [72]. SUMO1 (FC = 1.10, *p* = 0.042) encodes a smell ubiquitin-like modifier protein that has been shown to increase in models of AD [107] and of PD and ageing [164]. Functionally, increased levels of SUMO1 leads to hippocampal dysfunction, decreased cell proliferation, and reduced neuroblast differentiation [172]. In the context of neurodegenerative pathologies, SUMO1 overexpression leads to increased amyloid plaque density, increased dendritic spine loss, and more severe cognitive deficits in a mouse model of AD [73] and impairment of synaptic function associated with memory impairment [91]. Last in this group, TYROBP (FC = 1.15, *p* = 0.04) encodes a TREM2 adaptor protein and has been shown to be increased in AD patients and mouse models [17,^55^] as well as after TBI [18]. Overexpression of TYROBP can exacerbate tau-mediated neurodegeneration [133] and targeting of TYROBP in AD models is therapeutic, resulting in less severe neuritic dystrophy and attenuation of learning and behavioural impairment [85]. Together, these 10 upregulated genes suggest neurodegenerative-like changes in the brain 7d post-injury.

Three genes with increased expression levels suggest changes to largescale signalling pathways which likely have vast repercussions on brain function and health. MAPK10 (FC = 1.09, *p* = 0.05) encodes mitogen-activated protein kinase 10, which is a neuron-specific map kinase enzyme in the JNK signalling pathway. MAPK10 has been identified as a possible target and biomarker of neurodegenerative diseases [104]. Indeed, MAPK10 is associated with processes of ageing and neurological deficits [168] and enhances both amyloid beta production through the phosphorylation of amyloid precursor protein (APP) [74] and the maturation and development of neurofibrillary tangles [170]. MAPK10 has also been shown to mediate the death of dopaminergic neurons in PD [25]. RAB6B (FC = 1.14, *p* = 0.017) is also regulated by the JNK signalling pathway [178] and was significantly increased at 7d post-injury. RAB6B encodes a novel GTPase localized to microglia, pericytes, and Purkinje cells [113] where its role in the brain is not well established aside from its role in retrograde transport of cargo in neuronal cells [163]. PIK3CD (FC = 1.26, *p* = 0.005) encodes the phosphoinositide 3-kinase catalytic subunit which is a kinase in the PKA pathway associated with schizophrenia [63] and neurologically compromised brains [168]. PIK3CD is related to processes of ageing, and gain of function mutations in this gene result in increased number of senescent cells [88] and similarly inhibition of this PKA subunit is senolytic [183]. RB1CC1 (FC = 1.06, *p* = 0.041) encodes an autophagy-related protein for which expression increases in aged glial cells [139]. RB1CC1 activates the expression of the senescent factor p16 (or CDKN2A) [109], but is also an upstream regulator of autophagy [153]. Importantly, loss of autophagy signalling has been shown to cause deficiency in the DDR, particularly towards DSBs [86]. Together, these 3 upregulated genes indicate changes to large-scale signalling pathways including the JNK pathway, PKA pathway, and autophagy.

**Fig. 13:**
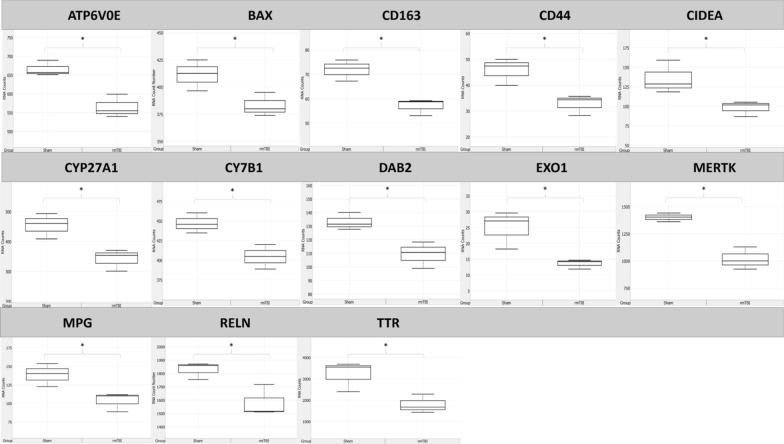
Significantly (*p* ≤ 0.05) downregulated genes in rmTBI mice compared to shams at 7d post-injury. Data are expressed as normalized RNA count number for shams and rmTBI. The median value is represented by the horizontal line within the box, defined by the first and third quartiles, and tails represent 1.5 × the interquartile range

Thirteen genes had significantly decreased expression in the ipsilateral hemisphere 7d post-injury (Fig. 13). Several of these expression signatures are consistent with reports on cellular senescence, neurodegenerative disease, and neuroinflammation resulting in barrier dysfunction.

BAX (FC = −1.07, *p* = 0.05) encodes a protein in the BCL2 protein family which regulates susceptibility to apoptosis [122, 169], being anti-apoptotic under low expression conditions, and which is known to decrease in senescent cells [37, 155]. In support of this anti-apoptotic mechanism, the apoptosis inducer CIDEA (FC = −1.38, *p* = 0.05) was also significantly decreased at this timepoint. Base-excision repair DNA repair and senescence-associated gene EXO1 (FC = −1.81, *p* = 0.04) was significantly decreased 7d post-injury. It has been reported that repression of EXO1 induces cellular senescence and, vice versa, that the activation of the p53 senescence pathway is sufficient to repress DNA repair activity [27]. SNPs in EXO1 have been associated with cognitive ageing [83], supporting its role in cellular senescence. MPG (FC = −1.34, *p* = 0.04) is also a DNA repair gene in the base-excision repair pathway. Its inhibition results in increased markers of senescence [142] and, similarly, its expression is decreased in senescent cells [4]. MERTK (FC = −1.38, *p* = 0.024) encodes a tyrosine kinase which is known to decrease in senescent cells and ageing [127], where it results in increased blood brain barrier permeability [98]. MERTK plays multiple roles in the cell, including mediating synapse remodelling and neural circuit refinement [26] and its dysregulation in AD has been suggested to contribute to chronic inflammation in AD pathology [69]. Inhibition of MERTK leads to cellular senescence in glioblastoma cells [146]. These 5 genes together suggest increased levels of cellular senescence at 7d post-injury.

Several downregulated genes at this timepoint reflect neurodegenerative changes. CYP27A1 (FC = −1.33, *p* = 0.03) encodes a cholesterol metabolizing enzyme in the brain, which when reduced leads to increased levels of 27-hydroxycholesterol, a feature of AD disease progression [148, 149] and learning and memory impairment [176]. 27-hydroxycholesterol has also been associated with impaired glucose metabolism in neurons [66]. CYP27A1 expression is reduced in neurons and around amyloid plaques in AD [13] and it is significantly reduced with physiological ageing [84]. CYP7B1 (FC = −1.10, *p* = 0.03) is another cholesterol metabolizing enzyme in the brain, for which loss results in increased oxysterol substrate accumulation and transport across the blood brain barrier [57]. CYP7B1 decreases with age [168] and its loss is associated with impaired spatial memory in rats [171]. RELN (FC = −1.16, *p* = 0.05) encodes the secreted extracellular matrix protein reelin, which plays important roles in controlling cell–cell interactions in the brain. RELN expression decreases early in AD prior to the deposition of amyloid pathology [59], and its loss is associated with cognitive deficits, several neuropsychiatric disorders, and loss of synapse maintenance [65]. Indeed, RELN expression is reduced in autism spectrum disorder [184] and schizophrenia and bipolar disorder where it associates with reduced dendritic spine density [50]. In addition, RELN plays a role in protecting against amyloid beta toxicity [76]. TTR (FC = −1.79, *p* = 0.04) encodes a carrier protein secreted by the choroid plexus endothelial cells into the CSF [180]. Reduced levels of TTR are seen in AD CSF and brain tissue [143]. Indeed, TTR plays a role in the clearance of amyloid peptides from the brain by binding to amyloid peptides and crossing the blood brain barrier specifically in the brain-to-blood direction exclusively to transport to the liver [1].

Consistent with some of the above changes, two downregulated genes suggest the presence of astrogliosis and possible dysfunction of critical brain barriers including the blood–brain barrier and the ependymal epithelial lining of the ventricles. CD44 (FC = −1.40, *p* = 0.03) is a marker of pro-inflammatory astrocytes, for which reduced expression is found in aged mice and in mice with neurological deficits [145]. In addition, CD44 deficient mice have significantly increased blood–brain barrier permeability which severely worsens the progression of multiple sclerosis [39]. DAB2 (FC = −1.22, *p* = 0.04) encodes a mitogen-responsive protein which plays a role in establishing epithelial cell polarity [99]. Notably, specialized epithelial cells in the ventricular lining of the brain called ependymal cells require maintenance of their polarity to regulate CSF dynamics [112, 141, 166].

At this timepoint two additional genes were significantly reduced: CD163 (FC = −1.26, *p* = 0.01), a macrophage marker, and ATP6V0E (FC = −1.18, *p* = 0.02), an ATPase transporter subunit for which its role in the brain is not well established.

**Fig. 14:**
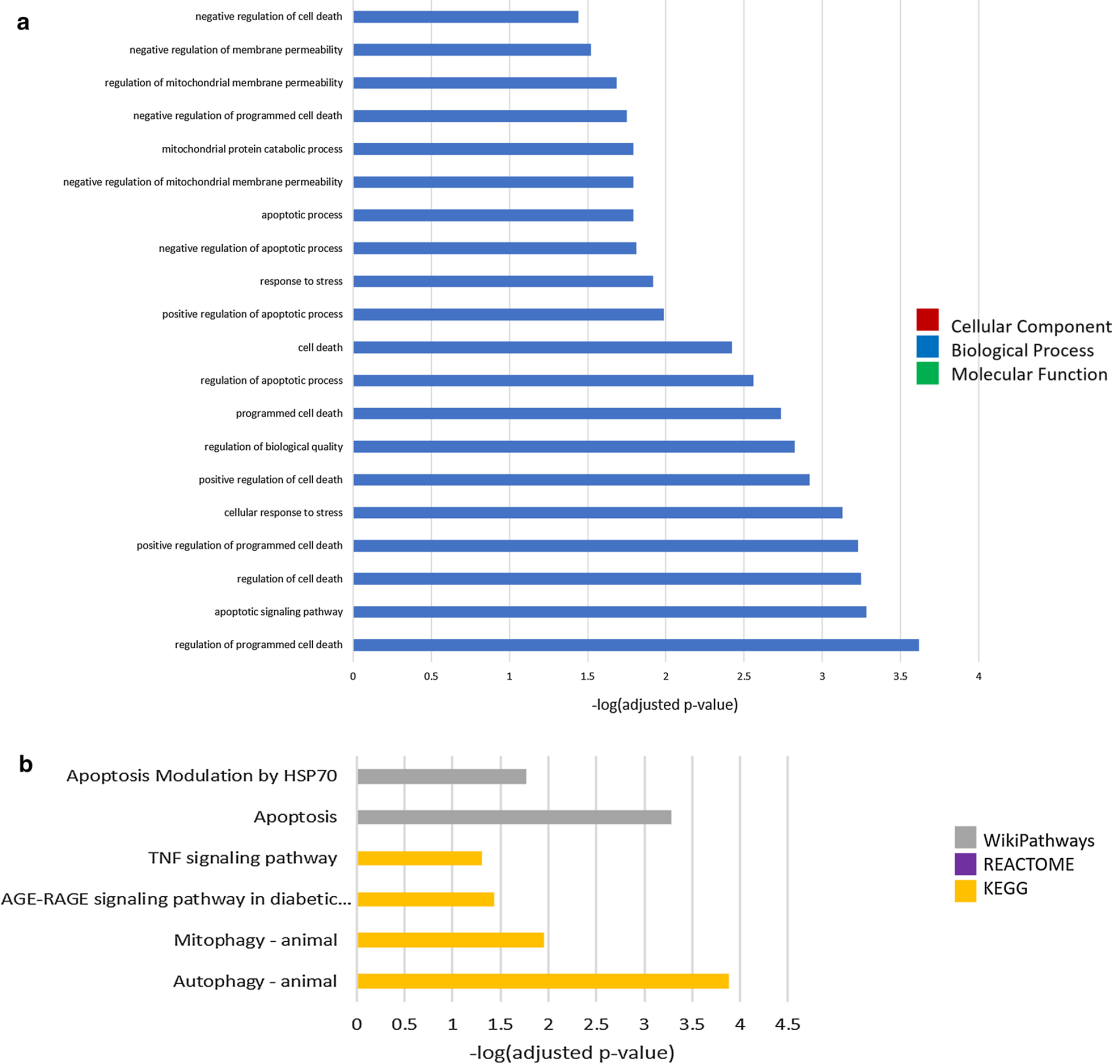
Gene ontology (**a**) and pathway (**b**) enrichment analyses of genes significantly (*p* ≤ 0.05) upregulated 7d post-injury

**Fig. 15:**
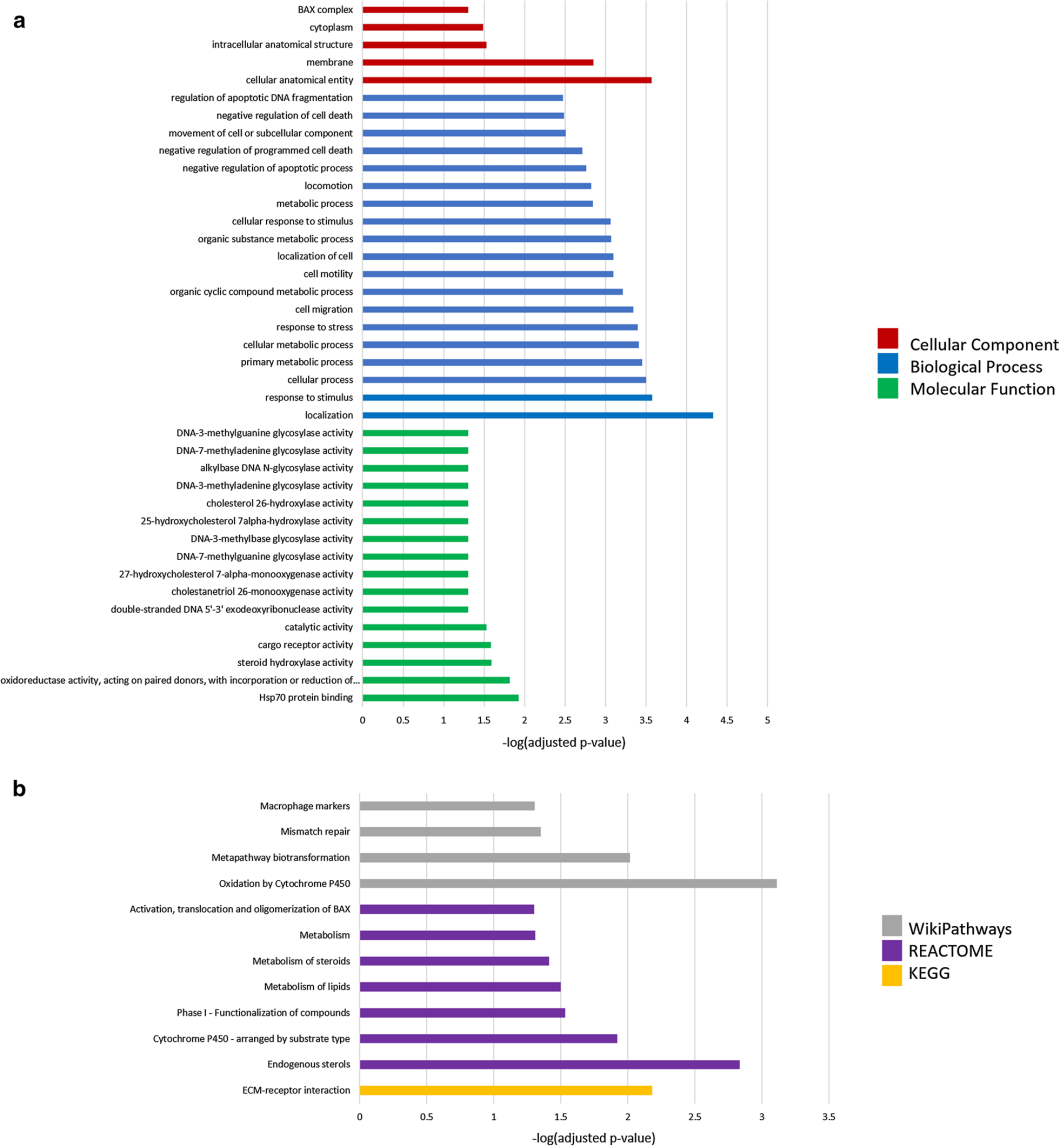
Gene ontology (**a**) and pathway (**b**) enrichment analyses of genes significantly (*p* ≤ 0.05) downregulated 7d post-injury

Gene ontology analysis of upregulated genes 7d post-injury revealed enrichment of 20 GO terms, all of which were biological process terms (Fig. 14a). These terms encompass regulation of cell death pathways, regulation of membrane permeability, regulation of the mitochondrial membrane, and the cellular response to stress. This GO enrichment analysis indicates that 7d post-injury the stress-response remains activated, and that significant regulation (both positive and negative) of cell death pathways is occurring. Similarly, pathway analysis of upregulated genes 7d post-injury (Fig. 15b) revealed enrichment of two terms from the WP database, apoptosis modulation by HSP70 and apoptosis, and four terms from the KEGG database, TNF signalling pathway, AGE-RAGE signalling pathway, mitophagy, and autophagy. This pathway analysis supports the dysregulation of cell death at this timepoint, with negative regulation suggested by apoptosis modulation by HSP70 and positive regulation suggested by apoptosis. Furthermore, this analysis suggests activation of the hypoxia-induced AGE-RAGE pathway (related to multiple age-related pathologies), and increased TNF, mitophagy, and autophagy signalling. These changes are consistent with the induction of cellular senescence at this timepoint.

Concomitantly, gene ontology analysis of downregulated genes 7d post-injury revealed enrichment of 40 terms (Fig. 15a), including 16 MF, 19 BP, and 5 CC terms. Enriched molecular function terms related primarily to the activity of various DNA glycosylases, cholesterol metabolizing enzymes, DNA exonucleases, and oxidoreductase and HSP70 activity. This analysis indicates a significant impairment of the DNA damage response, particularly the reduction of DNA damage repair enzymes, and impaired hypoxia response at 7d post-injury, which are consistent with the induction of cellular senescence. The enriched biological process terms encompassed regulation of cell death pathways, locomotion and metabolism, cell motility and migration, and the response to stress, suggesting significant changes in basic biological processes of the cell, perhaps due to the morphological changes associated with cellular senescence. Enriched cell component terms were the BAX complex, cytoplasm, intracellular anatomical structure, membrane, and cellular anatomical entity. Pathway analysis at 7d post-injury revealed enrichment of 12 terms (Fig. 15b). From the WP database, macrophage markers, mismatch repair, metapathway biotransformation, and oxidation by cytochrome P450 were significantly enriched. Similarly, from the Reactome database activation, translocation, and oligomerization of BAX, metabolism of steroids and lipids, functionalization of compounds, cyrochrome P450, and endogenous sterols were enriched. Lastly, ECM-receptor interaction was enriched from the KEGG database. Together, the pathway analysis at this timepoint supports reduction of the DDR, impaired oxidation reduction reactions, and impeded metabolism at this timepoint. These pathway changes support the roles of individual genes detailed above and suggests the induction of cellular senescence and neurodegenerative changes 7d post-injury.

### qPCR and Western Blot validation of targets

**Fig. 16:**
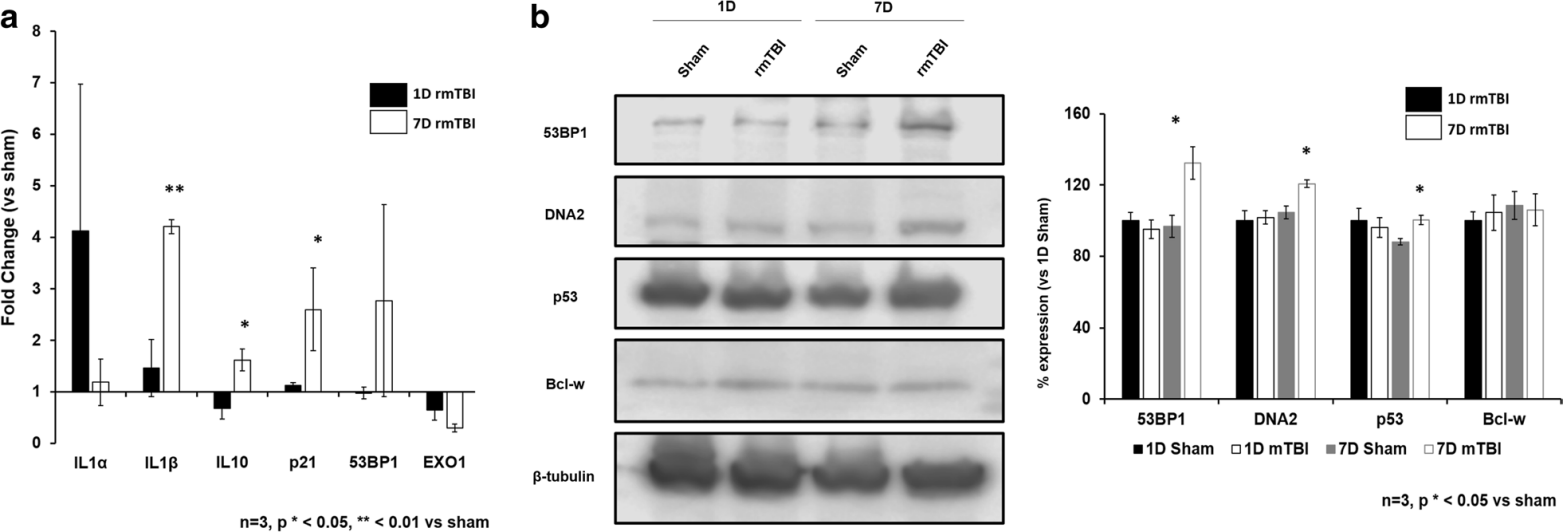
**a** qPCR and** b** Western Blot validation. Statistical analysis done in R using unpaired student t-test vs sham

We validated our findings using six qPCR and four WB targets related to DNA damage-induced cellular senescence. Using qPCR (Fig. 16a), we found elevation of IL1α at 1 day post-injury, which is a DNA damage sensing agent [125] and an upstream regulator of cellular senescence [114]. By 1 week post-injury, qPCR revealed significant elevation of senescence-associated interleukins IL1β (*p* = 0.04) and IL10 (*p* = 0.05), which promote senescence-mediated inflammation [77] and senescence induction through p53 and p21 [134], respectively. At 1 week post-injury qPCR also revealed elevation of the senescence marker p21, the DNA damage marker 53BP1, and EXO1, a DNA repair gene for which expression is reduced in senescent cells [27]. WB analysis supported these findings (Fig. 16b), showing elevation of the DNA damage-induced cellular senescence markers 53BP1 (*p* = 0.03), DNA2 (*p* = 0.01), and p53 (*p* = 0.03). These findings support and validate our gene expression data from NanoString, showing activation of the p21 and p53-dependent DNA damage-induced senescence pathways.

## Discussion

mTBI is associated with a long list of acute symptoms including, but not limited to, headaches, fatigue, anxiety, depression, attention deficits, short-term memory problems, insomnia, confusion, and irritability [90]. In addition to these acute changes, rmTBI can cause persistent symptoms for longer than 3 months, known as post-concussion syndrome [61], and an increased risk for several neurodegenerative diseases later in life [43]. There has been a focus in recent years on the characterization of the neuropathology of the injured brain, with CTE suggested as the pathological signature and driver of symptoms after mTBI [96]. While the risk of developing neurological disorders, including CTE, after repeated head injury is undeniable, it remains unclear what early pathophysiological mechanisms after mTBI may drive early symptoms and ultimately the toxic protein accumulation and neurodegeneration later in life. Furthermore, it also remains elusive which molecular changes drive the broad symptoms associated with mTBI both in the acute and chronic periods following injury.

Here we have presented a murine model of repeated mTBI. Histological analysis indicates absence of any gross visible lesion, yet progressively increased levels of gliosis and microglial activation at 1 day and 7 days post-injury and axonal damage at 7 days post-injury. These changes were accompanied by significant loss of righting reflex after each impact and impairment in the Morris water maze at 1 week post-injury. These changes are consistent with other studies using a closed skull model of mTBI, in which gliosis, microglial activation, and axonal damage are evident alongside neurobehavioural deficits [9].

Additionally, there are significant changes in numerous genes that point to DNA damage and early neurodegeneration as early as 24 h and ramping up after 7 days. 24 h following repeated injury, rmTBI mice had significantly increased expression of DSB repair enzymes in the DDR (ATG7, DNA2, NBN) and a marker of M2 macrophages (CD163), indicating the presence of neuroinflammation and DNA damage in the form of DSBs. At this timepoint rmTBI mice showed decreased expression of several cell death pathways including the membrane attack complex (C6), TNF-associated cell death (CASP9, TRADD, TRAF3), autophagy-mediated cell death (EPG5, MAPILC3A), and DNA methylation patterns (AGO4). At 24 h post-injury, therefore, the DDR and inhibition of cell death are evident, suggesting that cells are attempting to repair damage inflicted by the mTBI.

By 7d post-injury, many components of the DDR were significantly reduced (EXO1, MPG) with additional downregulation of cholesterol metabolizing enzymes (CUP27A, CYP7B1), loss of toxic protein clearance and other protective mechanisms against neurodegenerative mechanisms (MERTK, RELN, TTR, CD44, DAB2) and reduction of apoptosis (BAX, CIDEA). Up-regulated pathways at this timepoint include cellular senescence (AGO4, BCL2L2, BNIP3, BNIP3L, RAD51, RB1CC1), PKA and JNK signalling pathways (MAPK10, RAB6B, PIK3CD), and early neurodegenerative processes (CHST8, CLEC7A, DAPK1, GPR34, MAFB, MSR1, OLFML3, SUMO1, TYROBP). These results suggest that by 7d post-injury signs of brain senescence and neurodegeneration are detectable. These findings were supported with qPCR and WBs, directly showing elevation of IL1β, IL10, p21, 53BP1, DNA2, and reduction in EXO1—all of which are molecular signatures of cellular senescence induced by genotoxic stress.

Many studies using rodent models have been published characterizing the diverse secondary injury cascade that occurs in the acute period following mTBI. Neuroinflammation has been identified as a hallmark response in the acute (24 h to 7 days) period post-injury [87]. Indeed, various rodent models of mTBI have shown increased levels of infiltrating immune cells [34], reactive astrogliosis [12], microgliosis [12, 124], and increased levels of pro-inflammatory cytokines [131]. This acute period of neuroinflammation after injury has been shown to prime neurons towards a neurodegenerative phenotype in the long-term [102]. In addition to neuroinflammation, disruption of the blood brain barrier resulting in its increased permeability and changes in CSF dynamics has been reported in the acute period after mTBI in rodents [7, 47, 166]. These changes are frequently accompanied by behavioural changes suggestive of anxiety [12], depression [80], and cognitive impairment [12, 80]. Evidence of neuronal loss [14], diffuse axonal injury [36], and atrophy [53] are also evident in this acute period. Indeed, in this study we identified gliosis, microgliosis, and axonal damage at both 1 and 7 days post-injury. The gene expression changes found in this study support and reflect the findings presented above, but we have further identified DNA damage-induced cellular senescence as activated in the acute period following mTBI and a possible driver of these changes and of long-term effects of injury.

The results of this study are consistent with recently published mouse models of mTBI in which DNA damage has been reported in the early (24 h) period [3, 123, 151]. Our own studies on human autopsy cases showed strong evidence of DSBs in brains of athletes with a history of rmTBI [131, 132]. Activation of the DDR in response to DNA damage is essential for the maintenance of genome integrity [67]. Detection of the DDR is often lost once cellular senescence is induced [5, 40]. Concomitantly, the DDR is lost in ageing in association with the accumulation of senescent cells and “inflammaging” [111, 150]. IL1α was found to be elevated 1 day post-injury, an interleukin which senses DNA damage and is a regulator of the cellular senescence response to such genotoxic stress—yet by 1 week post-injury IL1α returns to baseline and signatures of senescence (namely elevated p21, p53, and reduced EXO1) are evident. Indeed, this mechanism is consistent with the gene expression and gene ontology analysis data presented here suggesting that by 7d post-injury the gene expression signature of rmTBI brains reflect cellular senescence and processes consistent with the aged brain.

A key finding in this study is the activation of neurodegenerative processes as early as 7d post-injury. Several significantly changed genes at this timepoint have been associated with AD-like pathology and disease progression (BCL2L2, CHST8, CLEC7A, CYP27A, CYP7B1, DAPK1, GPR34, MERTK, OLFML3, SUMO1, TTR, TYROBP), cognitive impairment (DAPK1, EXO1, RELN, SUMO1, TYROBP), PD progression (SUMO1, MAPK10), and the accumulation of toxic proteins (DAPK1, MAPK10, MSR1, OLFML3, SUMO1, TYROBP). In addition, several significantly changed genes at this timepoint play roles in the maintenance of critical brain barriers including the blood–brain barrier (CYP7B1, MERTK, CD44) and synapse maintenance in neuronal networks (MERTK, RELN), and associate with neurodegenerative disease pathogenesis in early stages prior to protein accumulation. Gene ontology and pathway analyses with GProfiler support these alterations. We therefore suggest that very early changes, namely DNA damage-induced cell senescence, after mTBI may predispose the brain to abnormal protein accumulation and neurodegenerative processes, due to a combination of increased pathogenic processes and a loss of protective mechanisms.

Gene expression changes reflective of early neurodegenerative processes in the acute period following rmTBI suggests that these processes may occur early and even prior to the accumulation of any abnormal neurotoxic protein species, such as hyperphosphorylated tau or amyloid beta. While these protein species are indisputably neurotoxic, we believe that they represent end-stage pathology and perhaps may not be the most optimal treatment target for preventing long-term symptoms after mTBI. Indeed, we suggest that the emergence of proteinopathy may be a result of some or all of these early pathogenic processes and loss of protective mechanisms and, because these changes occur very early, they may represent viable treatment targets for the prevention of long-term neurological sequalae after mTBI.

Decreased expression of critical DDR factors at 7d post-injury suggest that the brain may be more at risk in general to DNA damage in the acute period following mTBI. This poses an increased vulnerability for individuals who are at risk of repeated mTBI, such as professional athletes, military, or victims of domestic violence [2, 42, 121], who may face DNA damage from a subsequent brain injuries days or weeks apart and repeated many times over years. If the DDR is overwhelmed and consequently downregulated, subsequent damage from these injuries could hasten the progression of neurodegenerative conditions in these individuals. This may be why professional athletes, who experience head injuries frequently [29], commonly present with CTE or a combination of neurodegenerative pathologies at a significantly younger than average age [130]. Indeed, accumulation of DNA damage and defects in DNA repair machinery are linked to neurodegenerative mechanisms and severe neurological dysfunction. For example, mutations in the upstream DNA damage sensor protein ATM result in movement and coordination dysfunction, cerebellar atrophy, and speech impairments [8]. Similarly, mutations in the DNA repair enzyme Ligase 4 result in microcephaly [108, 115] and inactivation of the DNA repair factor PNKP has been shown to cause seizures, microcephaly, oligodendrocyte dysfunction, and mutations in this gene lead to cognitive impairments and even severe dementia [11]. Although a better understanding of the mechanisms linking DNA damage with neurological dysfunction and neurodegeneration are needed, there is sufficient evidence to suggest that the accumulation of DNA damage and, by extension, senescent cells, is sufficient to cause neurological symptoms and chronic pathology. Indeed, DNA damage-induced cellular senescence can lead to the induction of the SASP, in which pro-inflammatory secreted molecules contribute to chronic levels of neuroinflammation.

The gene expression signature at 7 days post-injury indeed suggests activation of DNA damage-induced cellular senescence pathways. This was validated on the tissue using immunohistochemistry for Lamin A/C, a nuclear lamin protein located adjacent to the inner nuclear membrane. Lamin A/C is a scaffolding protein important for the maintenance of genomic integrity through its chromatin stabilization role [70]. Loss of lamin A/C is associated with impaired DNA damage repair mechanisms [45] and induces cellular senescence characterized by defective DNA repair [119]. Hundreds of mutations in *LMNA*, the gene encoding Lamin A/C, have been identified as causing degenerative disorders, collectively called *laminopathies,* an example of which is Hutchinson–Gilford Progeria Syndrome (HGPS)*.* Molecularly, HGPS dysregulated gene expression, DNA repair defect, and premature accumulation of senescent cells [48, 148] Clinically, patients with HGPS exhibit signs of accelerated aging with a significantly reduced lifespan (mid-teens), severe atherosclerosis and cardiovascular pathology, hair loss, and reduced bone density [148]. Reduced expression of Lamin A/C in glial cells following rmTBI is therefore concerning, and suggestive of DNA damage-associated cellular senescence, perhaps contributing to neurological dysfunction.

It is important to note that not all individuals who experience repetitive brain injury have long-term symptoms nor are diagnosed with a neurodegenerative disease [58]. Indeed, the presentation of mTBI is extremely heterogeneous between individuals [92] and between sexes, where women tend to experience more severe symptoms [30]. We hypothesize that individual differences in reversing the DNA damage and related consequences may drive long term outcomes of brain injury. As an example, naturally occurring polymorphisms in DDR genes may confer different susceptibilities to mTBI. Consistent with this hypothesis, polymorphisms in the DNA repair gene EXO1, have been shown in humans to associate with cognitive ageing [83]. Susceptibility or vulnerability towards mTBI-induced DNA damage may therefore determine a patient’s long-term outcome following mTBI. In the context of sex differences, women have additional physiological levels of DNA damage in the brain, due to estrogen and estrogen metabolites [129], which may lower the threshold of what the DDR can handle before it is overwhelmed. Estrogen-induced DNA damage has further been associated with cognitive impairment [79], suggesting it plays a functional role in brain health. DNA repair pathways have previously been suggested as personalized medicine approaches for targeted cancer therapy, as several cancers present with defects in DNA repair machinery [32]. More research is warranted into the inter-individual and sex heterogeneity of the DDR in response to mTBI, as this represents a viable personalized medicine approach to early mTBI intervention.

## Conclusions

In this study, using a murine model of rmTBI we have shown evidence of a response to DNA damage in the brain 24 h following repeated brain injury, and progression to cellular senescence, impaired DNA damage responses, and brain ageing by 7 days. We suggest that these pathogenic processes are critical early events which may drive early symptoms as well as late onset neurodegenerative processes after mTBI they represent viable treatment targets for early intervention strategies. Within this strategy, personalized medicine approaches may be foreseeable due to naturally occurring polymorphisms and alterations in the DDR or hypoxia response which may underlie individual and sex differences.

## Supplementary Information

**Additional file 1.** NanoString data for 1-day post-injury group.

**Additional file 2.** NanoString data for 1-week post-injury group.

**Additional file 3: Table S1.** List of qPCR primers used in this study.

## Data Availability

The data supporting the conclusions of this article are included within the article’s additional files.

## Abbreviations

mTBI: Mild traumatic brain injury
rmTBI: Repeated mild traumatic brain injury
AD: Alzheimer’s disease
PD: Parkinson’s disease
ALS: Amyotrophic lateral sclerosis
FTD: Fronto-temporal dementia
CTE: Chronic traumatic encephalopathy
DSB: Double strand break
ROS: Reactive oxygen species
DDR: DNA damage response
SASP: Senescence-associated secretory phenotype
TCP: Centre for phenogenomics
FFPE: Formalin-fixed paraffin-embedded
FC: Fold change
GO: Gene ontology
KEGG: Kyoto Encyclopedia of genes and genomes
WP: WikiPathways
HDR: Homology-directed repair
NHEJ: Non-homologous end joining
SNP: Single nucleotide polymorphism
CSF: Cerebrospinal fluid
APP: Amyloid precursor protein
