# Supplementary figures and images for: Early onset senescence and cognitive impairment in a murine model of repeated mTBI
Source: Acta Neuropathol Commun. 2021 May 8;9:82. doi: 10.1186/s40478-021-01190-x (PMC8106230; doi:10.1186/s40478-021-01190-x)

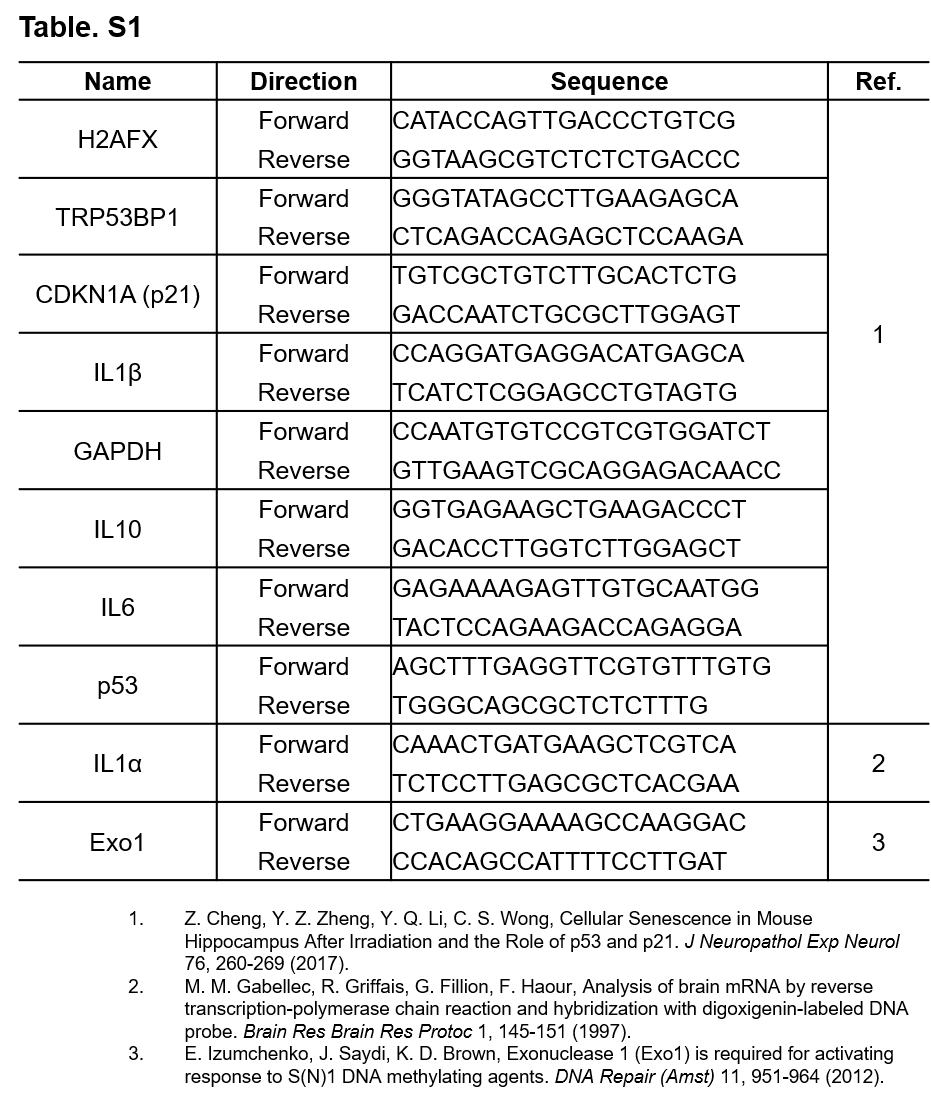

Supplement: Supplementary file 3 — Additional file 3: Table S1. List of qPCR primers used in this study. [file 40478_2021_1190_MOESM3_ESM.tif]
